# Negative Control of RpoS Synthesis by the sRNA ReaL in *Pseudomonas aeruginosa*

**DOI:** 10.3389/fmicb.2018.02488

**Published:** 2018-10-29

**Authors:** Hue Thi Bach Nguyen, David Romero A., Fabian Amman, Theresa Sorger-Domenigg, Muralidhar Tata, Elisabeth Sonnleitner, Udo Bläsi

**Affiliations:** ^1^Department of Microbiology, Immunobiology and Genetics, Max F. Perutz Laboratories, Center of Molecular Biology, University of Vienna – Vienna Biocenter, Vienna, Austria; ^2^Institute of Theoretical Chemistry, University of Vienna, Vienna, Austria

**Keywords:** *Pseudomonas aeruginosa*, ReaL, RpoS, translational silencing, small RNA

## Abstract

*Pseudomonas aeruginosa* (Pae) is an opportunistic human pathogen, able to resist host defense mechanisms and antibiotic treatment. In Pae, the master regulator of stress responses RpoS (σ^S^) is involved in the regulation of quorum sensing and several virulence genes. Here, we report that the sRNA ReaL translationally silences *rpoS* mRNA, which results in a decrease of the RpoS levels. Our studies indicated that ReaL base-pairs with the Shine-Dalgarno region of *rpoS* mRNA. These studies are underlined by a highly similar transcription profile of a *rpoS* deletion mutant and a *reaL* over-expressing strain.

## Introduction

*Pseudomonas aeruginosa* (Pae) is an opportunistic human pathogen that causes severe infections in immunocompromised individuals, burn patients, and patients suffering from cystic fibrosis. Owing to its ability to form biofilms, Pae can resist host defense mechanisms and antibiotic treatment (Costerton et al., 1999). Many Pae virulence factors are controlled by numerous two component systems, extra-cytoplasmic sigma factors, and by the four inter-linked quorum sensing (QS) systems Las, Rhl, Pqs, and Iqs (Van Delden and Iglewski, 1998; Lee and Zhang, 2015).

Different sigma factors control different subsets of genes, enabling bacteria to adapt to environmental changes (Wosten, 1998). While expression of housekeeping genes is regulated by σ^70^, Pae encodes a number of additional sigma factors (Martínez-Bueno et al., 2002), one of which is RpoS (σ^S^), considered to be the master regulator of stress responses. RpoS is predominantly expressed during stationary growth (Fujita et al., 1994) and under other stress conditions, including low pH, heat shock, oxidative stress, and increased osmolarity (Jorgensen et al., 1999; Cochran et al., 2000; Venturi, 2003; Schuster et al., 2004; Dong and Schellhorn, 2010). As RpoS controls the synthesis of quorum-sensing (QS) dependent response regulators in Pae, RpoS governs the expression of a large subset of genes including functions required for the synthesis of virulence factors such as pyocyanin, exotoxin A, LasA and LasB elastases, and exoenzyme S (Suh et al., 1999; Sonnleitner et al., 2003; Hogardt et al., 2004).

Bacterial sRNAs play a major role in post-transcriptional regulation. Their expression is usually induced by environmental cues and leads to a fast response and adaptation to stressors and/or habitats (Hoe et al., 2013). In general sRNAs modulate gene expression in a positive- or negative manner by base-pairing with their target mRNAs, which often requires the RNA chaperone Hfq (Storz et al., 2011). In addition, RNAs can mediate gene expression by sequestration of regulatory proteins, as exemplified by the Pae RNAs CrcZ (Sonnleitner and Bläsi, 2014) and RsmW/RsmY/RsmZ (Lapouge et al., 2008; Miller et al., 2016). While numerous candidate sRNAs have been identified in several Pae strains (Livny et al., 2006; Sonnleitner et al., 2008; Sonnleitner and Haas, 2011; Ferrara et al., 2012; Gómez-Lozano et al., 2012; Wurtzel et al., 2012; Caldelari et al., 2013) only a few, including the sRNAs PhrS (Sonnleitner et al., 2011), NrsZ (Wenner et al., 2014), PrrF1-2 (Reinhart et al., 2015), ErsA (Ferrara et al., 2015), PesA (Ferrara et al., 2017), sr0161, and Sr006 (Zhang et al., 2017), have been functionally characterized.

In *E. coli*, the expression of *rpoS* is predominantly regulated post-transcriptionally. The *E. coli* 5′ untranslated region (UTR) of *rpoS* mRNA contains a stem-loop structure that occludes the ribosome binding site (RBS) (Majdalani et al., 1998). Binding of sRNAs to this region disrupts the secondary structure and results in enhanced *rpoS* mRNA translation, which is accompanied by stabilization of the mRNA (Majdalani et al., 1998; Majdalani et al., 2002; McCullen et al., 2010). In *E. coli*, the three Hfq-binding sRNAs, DsrA, RprA, and ArcZ, are known to activate *rpoS* mRNA translation through this mechanism (Majdalani et al., 1998; Mika and Hengge, 2014), whereas OxyS RNA represses RpoS synthesis by a mechanism that is not fully understood (Zhang et al., 1998). Similarly, as observed for OxyS RNA in *E. coli*, ectopic expression of the RpoS-dependent sRNA RgsA decreased RpoS synthesis in a Hfq-dependent manner during exponential growth phase in Pae (Lu et al., 2018). However, the latter authors did not obtain evidence for base-pairing between *rpoS* mRNA and RgsA. In addition, the physiological implications for these findings remain unclear as RgsA did not impact on RpoS synthesis during stationary growth, when the increase in RpoS levels results in increased *rgsA* expression (Lu et al., 2018).

Carloni et al. (2017) characterized the Pae PA14 sRNA SPA0084 (Ferrara et al., 2012) as a QS regulator that is up-regulated during stationary growth. They termed the sRNA ReaL for ‘regulator of 2-alkyl-4(1H)–quinolone′. The *reaL* gene, which is highly conserved among 13 Pae strains, is located in Pae strain PAO1 in the intergenic region between the two open reading frames PA3535 and PA3536 (Carloni et al., 2017; Supplementary Figure S1A). The analysis of the promoter region of *reaL* revealed a consensus motif for s^70^ and RpoS, respectively (Carloni et al., 2017). The RpoS dependency was supported by the finding that ReaL synthesis did not increase during stationary phase in a PA14 *rpoS* deletion mutant (Carloni et al., 2017). It was further shown that ReaL acts as a link between the Las and PQS systems. Carloni et al. (2017) reported that LasR negatively regulates *reaL* transcription and that ReaL interacts with and positively regulates *pqsC* translation in a Hfq-independent manner, which finally results in increased PQS production and in the establishment of PQS dependent virulence traits such as pyocyanin production, biofilm formation, and swarming motility. These findings could be reconciled with an attenuated and a hyper-virulent phenotype of a *reaL* deletion strain and a *reaL* overexpressing strain, respectively.

The Pae sRNA SPA0084/ReaL has been independently identified in our laboratory as PaeIII (Sorger-Domenigg, 2010). A comparative RNA_Seq_ based transcriptome analysis of a *reaL* overproducing PAO1 strain and a PAO1 *reaL* deletion strain revealed striking similarities with the expression profile obtained with a PAO1 *rpoS* deletion strain (Schuster et al., 2004). Using sRNA target identification by ligation (Han et al., 2016) and *rpoS::lacZ* translational reporter gene fusions, we provide evidence that ReaL directly represses *rpoS* mRNA translation through base-pairing with a sub-sequence encompassing the Shine and Dalgarno sequence of the latter. Hence, we identify ReaL as the first bacterial sRNA that translationally silences *rpoS* mRNA through a base-pairing mechanism.

## Materials and Methods

### Bacterial Strains and Growth Conditions

Strains and plasmids used in this study are listed in Supplementary Table S1. Unless indicated otherwise, the cultures were grown aerobically at 37°C in Luria–Bertani (LB; Miller, 1972) broth supplemented with appropriate antibiotics.

The PAO1Δ*reaL* strain was constructed through homologous recombination as previously described (Ye et al., 1995). Briefly, the upstream and downstream sequences of *reaL*, spanning the regions -655 to -26 and +62 to +666 with regard to the transcriptional start site of *reaL*, were amplified from PAO1 chromosomal DNA by PCR using primer combinations V34/W34 and Q32/R32 (Supplementary Table S2), respectively. The 629 nt upstream PCR product was cloned into plasmid pSUP202 using the restriction sites *Pst*I and *Pvu*I to generate pSUP202-ReaLup. The 604 nt downstream PCR product, containing the *reaL* rho-independent terminator sequence, was then cloned into plasmid pSUP202-ReaLup using the restriction sites *Pvu*I and *Eco*RI, resulting in the generation of plasmid pSUP202-ReaLko. This plasmid (suicide vector) was transformed into the *E. coli* strain S17-1 and then transferred by conjugation to PAO1. The plasmid was chromosomally integrated by homologous recombination in the presence of tetracycline. The excision of the vector by a second crossover event was achieved by enrichment for tetracycline-sensitive cells (Ye et al., 1995). The chromosomal deletion of *reaL* was verified by DNA sequencing and Northern-blot analysis.

### Construction of Plasmids

The plasmids for over-expression of *reaL* and the *reaL* variant harboring a deletion spanning nt +11 to +18 with respect to its transcriptional start site were constructed as follows. Full length *reaL* as well as the *reaL*_Δ11-18_ variant were amplified from PAO1 chromosomal DNA by PCR using primer pairs Y138/Z138 and E163/Z138 (Supplementary Table S2), respectively. The PCR fragments were then cloned into the *Xba*I, and *Pst*I sites of plasmid pKH6. In the resulting plasmids, pKH6-ReaL and pKH6-ReaL_Δ11-18_, *reaL* and *reaL*_Δ11-18_ are under the transcriptional control of an arabinose-inducible P_BAD_ promoter.

A translational *rpoS::lacZ* fusion under the transcriptional control of the *rpoS* promoter was constructed as follows. The *rpoS* promoter and the first 56 *rpoS* codons were amplified by PCR using oligonucleotides A52 and B52 (Supplementary Table S2), which contained a *Bam*HI and a *Pst*I restriction site, respectively. The PCR product was cloned into the corresponding restriction sites of plasmid pME6014 to generate plasmid pME6014-RpoS. This PCR product was also cloned into the corresponding restriction sites of plasmid pME6016 to generate plasmid pME6016-RpoS, harboring a transcriptional *rpoS-lacZ* reporter gene.

### RNA Isolation

Total RNA was isolated using the hot phenol method as described by Leoni et al. (1996). Briefly, the cells were harvested by centrifugation. The pellet was resuspended in 50 μl DEPC treated ddH_2_O and mixed with pre-heated (65°C) 250 μl lysis buffer and 500 μl phenol (pH 5.5) and vigorously vortexed. After centrifugation (5 min at 16,000 *g*), the aqueous phase was extracted with an equal volume of phenol/chloroform (1:1), re-centrifuged, and then extracted with an equal volume of chloroform. The RNA was precipitated over-night at -20°C, by addition of 0.1 volume of 3 M Na-acetate (pH 5.5) and 2.5 volumes of 96% ethanol. Removal of DNA was achieved by multiple rounds of TURBO DNase I (ThermoFisher) treatment followed by phenol chloroform extraction and Na-acetate/ethanol precipitation.

### Mapping of the ReaL Termini

Simultaneous mapping of the 5′ and 3′ ends of ReaL by RACE (Rapid Amplification of cDNA Ends) using circularized RNAs was performed as previously described (Toledo-Arana et al., 2009). Briefly, 6 μg total RNA were treated with DNase I (Roche) followed by phenol/chloroform extraction. The RNA was divided into two aliquots, one of which was treated with tobacco acid pyrophosphatase, (TAP) (Epicentre Biotechnologies) as specified by the manufacturer. After TAP treatment, the RNA was extracted with acid–phenol/chloroform followed by ethanol precipitation. Serial dilutions (from 500 to 0.5 ng) of the TAP+ and TAP- RNAs were prepared. Each dilution was ligated with 40 U T4 RNA ligase I (Fermentas) in the presence of 1× RNA ligase buffer in a total volume of 25 μl at 37°C for 1 h. The RNA was again extracted with acid–phenol/chloroform and precipitated with ethanol. The RNAs were resuspended in 10 μl RNase-free water. The subsequent RT-PCR reactions were performed with the oligonucleotides B53 and C53 (Supplementary Table S2) and the SuperScript One-Step RT-PCR kit (Invitrogen). DNA fragments of the expected size present in the TAP+ reactions were gel extracted, purified, and then cloned into the pGEM-T Easy Vector System (Promega). Five plasmid clones were sequenced and the sequences were compared to the PAO1 genome to localize the 5′- and 3′-ends of ReaL.

### RNA-Sequencing

PAO1Δ*reaL*(pKH6) and PAO1Δ*reaL*(pKH6-ReaL) were grown in LB medium supplemented with 50 μg/ml gentamycin until they reached an OD_600_ of 2.5. ReaL synthesis was induced with L-arabinose (final concentration 0.2%). After 30 min, total RNA was isolated from three biological replicates of either strain as described above. Ribosomal RNA was depleted with the Ribo-Zero rRNA Removal Kit (Illumina). Then, libraries were prepared using the NEBNext Ultra Directional RNA Library Prep Kit (Illumina) and sequenced using the Illumina HiSeq 2000, 100 bp single end read platform at the Vienna Biocentre Core Facilities.^1^ After removal of the adaptors, the sequences were quality trimmed with trimmomatic using default parameters (Bolger et al., 2014). The resulting sequences were then mapped onto the PAO1 reference genome (NC_002516.2) using Segemehl (Hoffmann et al., 2009, 2014) with default parameters. To allow sequence visualization in the UCSC Genome Browser (Chan et al., 2012), the ViennaNGS toolbox (Wolfinger et al., 2015) was used. For differential gene expression analysis, reads were counted using BEDtools (Quinlan and Hall, 2010) and analyzed using the DESeq2 package (Love et al., 2014). All RNAs with a log_2_-fold change greater than ±1.5 and a multiple testing adjusted *p*-value below 0.05 were considered to be differentially abundant. The raw sequencing data were deposited in the European Nucleotide Archive (ENA) as a study under the accession number PRJEB28696.

### Northern-Blot Analysis

The abundance of ReaL was determined by Northern blotting using 10 μg total RNA. The RNA samples were denatured for 5 min at 65°C in loading buffer containing 50% formamide, separated on 8% polyacrylamide/8 M urea gels, and then transferred to nylon membranes by electroblotting. The RNAs were cross-linked to the membrane by exposure to UV light. The membranes were hybridized with gene-specific ^32^P-end-labeled oligonucleotides (ReaL: W28; 5S rRNA: I26), and the hybridization signals were visualized using a PhosphorImager (Molecular Dynamics).

### Determination of the Steady State Levels and Half-Life of *rpoS* mRNA by RT-qPCR in the Presence and Absence of ReaL

PAO1Δ*reaL*(pKH6) and PAO1Δ*reaL*(pKH6-ReaL) were grown in LB medium supplemented with 50 μg/ml gentamycin until they reached an OD_600_ of 2.5. ReaL synthesis was induced with L-arabinose (final concentration 0.2 %). After 30 min (t_0_), the first sample was withdrawn and rifampicin was added to a final concentration of 250 μg/ml. Additional samples were then taken after 1, 2, 5, and 7.5 min, respectively. Then, total RNA was isolated from either sample as described above and DNase I treated. cDNA was synthesized from 2 μg of DNA-free total RNA using random hexamer primers (Promega) and AMV reverse transcriptase (Promega) according to the instructions of the manufacturer. For qPCR, 100 ng cDNA was used as template from either sample. The qPCR was performed with *rpoS* specific primers J98 and K98. The transcript levels of the *rpoD* gene obtained with the primers Q117 and R117 were used for normalization of the t_0_ samples as described by Lee et al. (2012). The real-time PCR mixture containing 5× HOT FIREPol EvaGreen qPCR Mix (Medibena), 100 ng cDNA, and 250 nM of each primer was placed in a Real-time PCR cycler (Eppendorf Mastercycler), and the reaction was started at 95°C for 5 min, followed by 40 cycles of 20 s at 95°C, 20 s at 55°C, and 20 s at 72°C. For all reactions including the DNA standards and the negative control (no template), two biological replicates and three technical replicates each were performed. The fluorescence was measured at the last step of each cycle. After 40 cycles, a melting curve analysis was performed by raising the temperature from 45°C to 95°C every 15 s, and by measuring the fluorescence at each cycle. The melting curve analysis yielded a single peak in the melting curve. Changes in the *rpoS* levels were estimated as previously described (Pfaffl, 2001).

### Western-Blot Analysis

The cells were cultured in LB broth until they reached an OD_600_ of 1.0. Then, ReaL synthesis was induced with L-arabinose (0.2% final concentration), and the cells were cultured for another 30 min. Aliquots containing cells from a total OD_600_ of 2 were harvested by centrifugation at 15,500 *g* for 2 min, resuspended in 100 μl Laemmli buffer, and lysed by heating to 95°C for 5 min. The cell lysates were then centrifuged at 26,000 *g* for 25 min and the pellets were discarded. A 10-μl aliquot of each sample was separated on a 10% SDS-polyacrylamide gel, and the proteins were transferred to a nitrocellulose membrane (Amersham Protran, GE Healthcare Life Sciences) by electroblotting. The membranes were incubated for 1 h at room temperature in TBST [25 mM Tris (pH 7.4), 150 mM NaCl, 0.05% Tween-20] containing 2.5% non-fat dry milk followed by washing in TBST. After washing, the membranes were probed at 4°C overnight with anti-RpoS antibodies. Probing with anti-ribosomal protein S1 antibodies served as a loading control. The membranes were washed in TBST and incubated with a goat anti-rabbit IgG horseradish peroxidase-linked antibody (Cell Signaling Technology, Europe) for 1 h at room temperature. Finally, the membranes were washed with TBST, and the proteins were detected with the enhanced chemiluminescence detection kit (Thermo Fisher Scientific, Inc.). Protein levels were calculated using the ImageLab work-suite (Bio-rad) and normalized against ribosomal protein S1 (internal control).

### Modified GRIL-Seq

To corroborate the ReaL-*rpoS* interaction, the GRIL-seq approach (Han et al., 2016) was employed. Briefly, the strains PAO1Δ*reaL* (pKH-ReaL; pKH13-t4rnl1) and PAO1Δ*reaL* (pKH6; pKH13-t4rnl1) were cultured in 15 ml LB broth supplemented with 50 μg/ml gentamicin and 250 μg/ml carbenicillin until they reached an OD_600_ of 1.8. T4 RNA ligase gene expression was induced with IPTG to a final concentration of 1 mM. After 1 h, ReaL synthesis was induced with L-arabinose (0.2% final concentration), and the cells were cultured for another 30 min. Then, the cells were harvested by centrifugation at 12,000 *g* for 5 min and total RNA was isolated as described above. Next, reverse transcription was performed with AMV reverse transcriptase (Promega), 1 μg total RNA, and the *rpoS* specific primer Z144 (Supplementary Table S2). ReaL-*rpoS* chimera were amplified by PCR using primers Y144 and Z144 (Supplementary Table S2), and a 1-μl aliquot of the reverse transcription reaction as template. The PCR products were purified using a Gel Extraction kit (QIAGEN) and cloned into the *SmaI* site of plasmid pUC19. Finally, the inserts were sequenced using the universal sequencing primer M13 (Supplementary Table S2).

### β-Galactosidase Assays

Briefly, PAO1Δ*reaL*(pKH6; pME6014-RpoS), PAO1Δ*reaL*(pKH6-ReaL; pME6014-RpoS), PAO1Δ*reaL*(pKH6-ReaL_Δ11-18_; pME6014-RpoS), PAO1Δ*reaL*(pKH6; pME6016-RpoS), and PAO1Δ*reaL*(pKH6-ReaL; pME6016-RpoS) were grown in triplicates in 20 ml LB broth. At an OD_600_ of 2.5, the synthesis of ReaL or of the ReaL_Δ11-18_ variant was induced with L-arabinose (0.2% final concentration). Cells were cultured for another 30 min after which samples were withdrawn. Then, the β-galactosidase activities were determined from equal volumes of cells as described (Miller, 1972).

## Results

### Negative Regulation of RpoS Dependent Genes by ReaL

To identify novel sRNAs in PAO1, an *in silico* search was performed using the general scheme described by Lenz et al. (2004). This screen revealed the putative sRNA PaeIII (Sorger-Domenigg, 2010), which was later also identified with an RNA_Seq_ approach as SPA0084 (Ferrara et al., 2012), and recently renamed ReaL (Carloni et al., 2017). The expression of *reaL* in strain PAO1 was confirmed by Northern blot analysis (Supplementary Figure S1B). The transcriptional start site of *reaL* in sPAO1 was mapped to genome position 3958053 by a 5′-3′ circularization approach (Supplementary Figure S1C). Based on this information, a PAO1Δ*reaL* strain was generated and transformed with either the *reaL* encoding plasmid pKH6-ReaL or the parental vector pKH6. As shown by Northern-blot analyses, ReaL was synthesized in PAOΔ*reaL*(pKH6-ReaL) and was absent in PAO1Δ*reaL*(pKH6) (Supplementary Figure S2).

**FIGURE 1:**
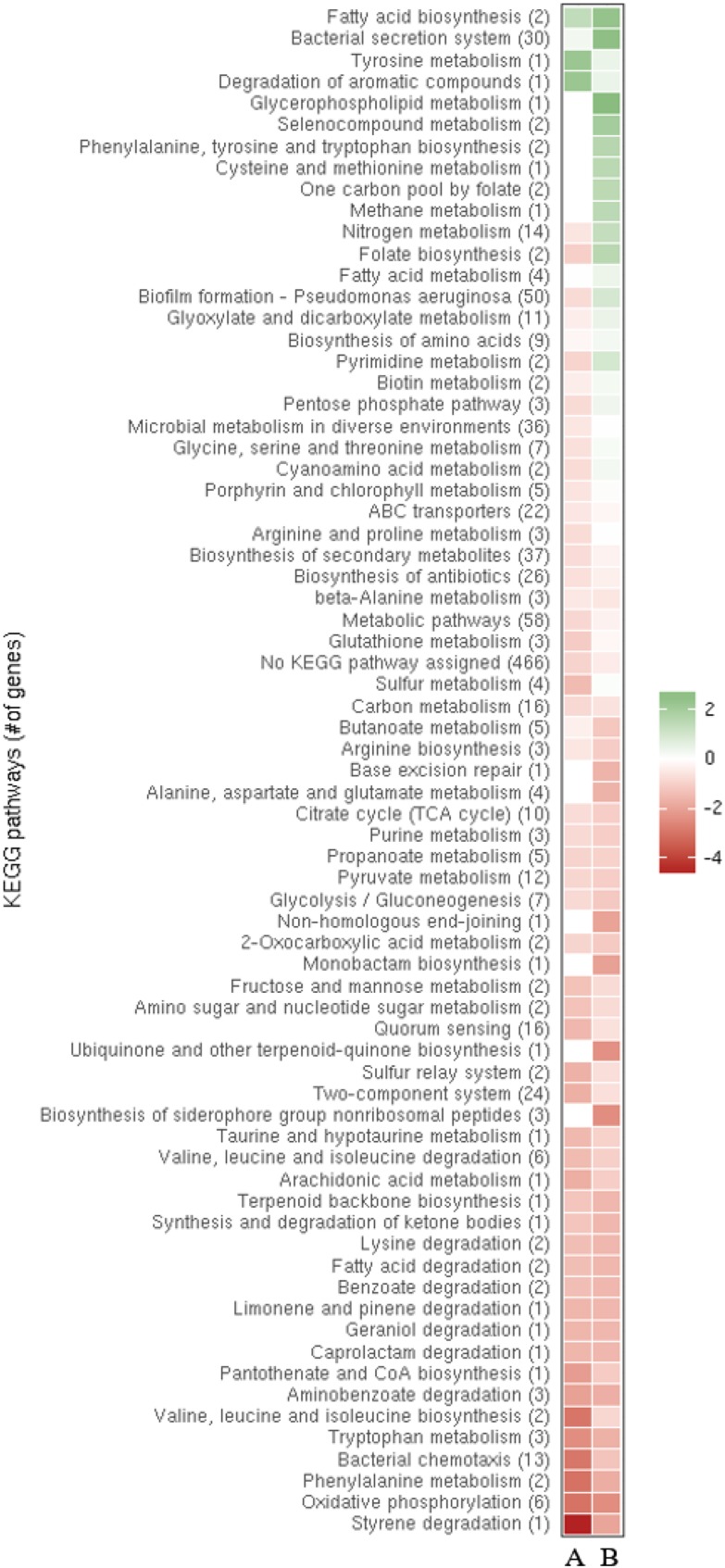
Meta-analysis of normalized expression of differentially abundant transcripts in strains PAO1Δ*reaL*(pKH6-ReaL) versus PAO1Δ*reaL*(pKH6) and strains PAO1D*rpoS* versus PAO1 (Schuster et al., 2004). The genes are grouped into the corresponding pathways (http://www.kegg.jp/kegg-bin/show_organism?org=pau). The columns denote PAO1Δ*reaL*(pKH6-ReaL) versus PAO1Δ*reaL*(pKH6) **(A)** and PAO1Δ*rpoS* versus PAO1 **(B)**, respectively. The color code shown in the scale at the right denotes log_2_-fold changes and fold change for the RNA_Seq_-based analysis, and the microarray-based analysis, respectively. Red indicates an overall decrease and green indicates an overall increase in the transcript levels of genes in a particular pathway. The numbers of genes within each pathway are indicated by the numbers given in parenthesis.

To study the impact of ReaL on global transcription, a comparative RNA_Seq_ based transcriptome analysis was performed with PAO1Δ*reaL*(pKH6-ReaL) and PAO1Δ*reaL*(pKH6). A *p*-value (adjusted for multiple testing) of 0.05 was set as a threshold for significance and the change in abundance for a given transcript had to exceed a log_2_ ± 1.5-fold change to be considered differentially abundant (Supplementary Table S3). In brief, the largest differential abundance was observed for transcripts whose functions are involved in anaerobic respiration, in the synthesis of fimbriae, carbon source utilization, production of siderophores, and other virulence factors, many of which are regulated by RpoS (Schuster et al., 2004; Supplementary Table S3). In fact, the transcriptional profile of the PAO1Δ*reaL*(pKH6-ReaL) strain resembled that previously described for a PAO1Δ*rpoS* strain at the level of individual transcripts (Schuster et al., 2004). Next, a meta-analysis of normalized expression of differentially abundant transcripts was performed for the following strain pairs: PAO1Δ*reaL*(pKH6-ReaL) versus PAO1Δ*reaL*(pKH6) and PAO1Δ*rpoS* versus PAO1. For this purpose, the genes were grouped into the corresponding KEGG pathways.^2^ As shown in the heat-map, the transcriptional profiles of the ReaL over-producing strain and the *rpoS* deletion strain were closely related at the level of the KEGG pathways (Figure 1). Very similar changes in the abundance of transcripts assigned to distinct pathways were observed for PAO1Δ*reaL*(pKH6-ReaL) and PAO1Δ*rpoS* when compared with strains PAO1Δ*reaL*(pKH6) and PAO1, respectively (Figure 1). In summary, these analyses suggested that ReaL might negatively impact RpoS synthesis.

### ReaL Interacts With *rpoS* mRNA

**FIGURE 2:**
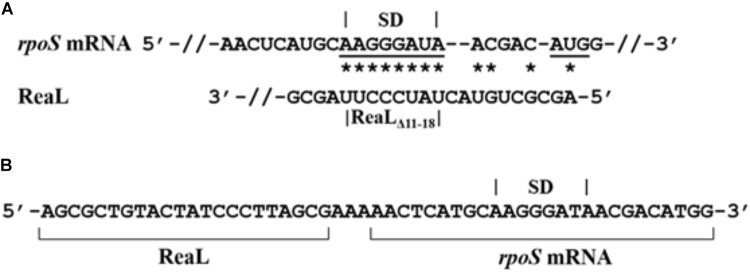
Interaction of ReaL with the TIR of *rpoS* mRNA. **(A)** Complementarity of ReaL with the TIR of *rpoS* mRNA predicted with the IntaRNA algorithm (Mann et al., 2017). Possible base-pairing interactions are denoted by stars. The *rpoS* SD region and the start codon are underlined, respectively. The deletion of nt 11–18 present in ReaL_Δ11-18_ is indicated below. **(B)** ReaL-*rpoS* mRNA RLM-RT-PCR. The sub-sequence generated by ligation of ReaL with *rpoS* mRNA is shown. The ReaL and *rpoS* mRNA sequences are indicated by brackets and the *rpoS* SD region is denoted on top.

As judged from the RNA_Seq_ analysis, the transcript levels of *rpoS* were ∼19-fold decreased in strain PAO1Δ*reaL*(pKH6-ReaL) when compared with PAO1Δ*reaL*(pKH6) (Supplementary Table S3) 30 min after induction of the *reaL* gene, which could be a consequence of translational silencing of *rpoS* mRNA by ReaL followed by degradation of the mRNA. Similarly, qPCR revealed a ∼18-fold decrease in the *rpoS* levels under the same experimental conditions (Supplementary Figure S3). In addition, the half-life of *rpoS* mRNA was ∼fivefold reduced in the ReaL overproducing strain when compared with the control strain (Supplementary Figure S3). Therefore, an *in silico* approach (IntaRNA) (Mann et al., 2017) was first used to explore whether ReaL might interact with the translation initiation region (TIR) of *rpoS* mRNA. This analysis suggested an interaction between the first 18 nt of ReaL and the TIR of *rpoS* (Figure 2A).

To corroborate the predicted ReaL-*rpoS* mRNA interaction *in vivo*, RNA ligase-mediated RT-PCR (RLM-RT-PCR) based on the GRIL-seq approach (Han et al., 2016) was employed. Briefly, GRIL-seq uses the ability of T4 RNA ligase to link two base-paired RNA molecules *in vivo*. In case of base-pairing between a sRNA and its target RNA(s), chimeras of the sRNA and the target RNA(s) are generated. These chimeras are then reverse transcribed, amplified, and sequenced to identify targets for a given sRNA. The strains PAO1Δ*reaL*(pKH6) and PAO1Δ*reaL*(pKH6-ReaL) were additionally transformed with plasmid pKH13-t4rnl1, encoding the T4RNA ligase gene. ReaL and T4RNA ligase synthesis was induced with IPTG and L-arabinose, respectively. Then RLM-RT-PCR was performed with total RNA isolated from either strain, using *rpoS* and *reaL* specific oligonucleotides (Supplementary Table S2). A product of ∼200 nt in length was detected in total RNA isolated from strain PAO1Δ*reaL*(pKH6-ReaL; pKH13-t4rnl1), whereas this product was absent in total RNA isolated from strain PAO1Δ*reaL*(pKH6; pKH13-t4rnl1). Cloning and sequencing of the ∼200 nt product detected in PAO1Δ*reaL*(pKH6-ReaL; pKH13-t4rnl1) confirmed the formation of a ReaL-*rpoS* chimera *in vivo* (Figure 2B). This chimera contained the first 22 nt of ReaL, an adenosine triplet followed by the sub-sequence of the *rpoS* mRNA predicted to interact with ReaL.

### ReaL Inhibits RpoS Synthesis

**FIGURE 3:**
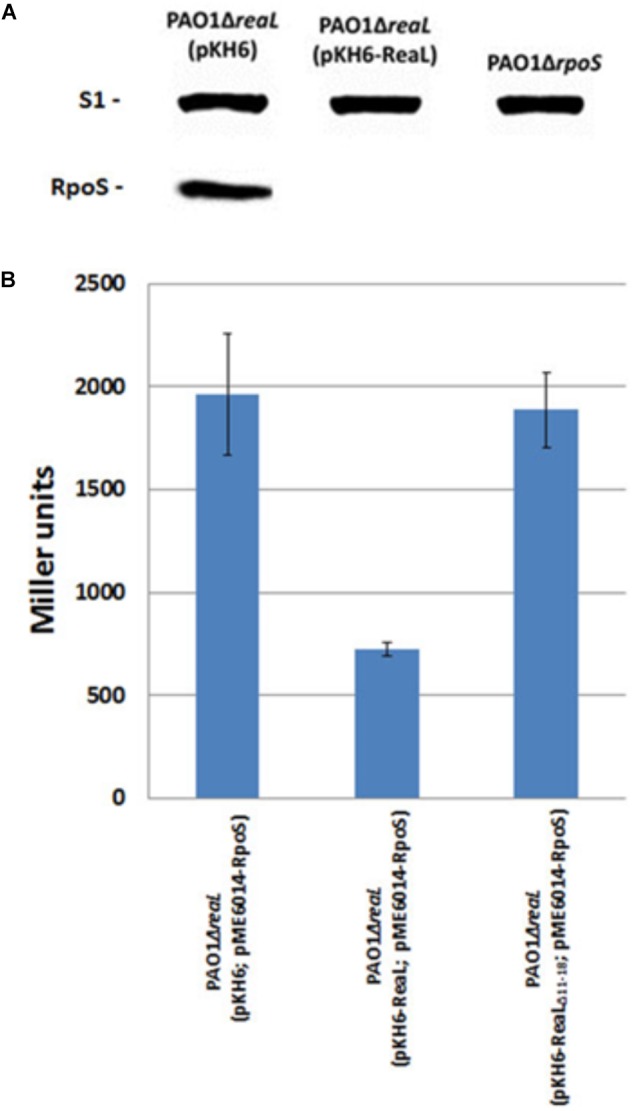
RpoS synthesis is repressed by ReaL. **(A)** The strains PAO1Δ*reaL*(pKH6), PAO1Δ*reaL*(pKH6-ReaL), and PAO1Δ*rpoS* were grown in LB medium. At an OD_600_ of 1, ReaL synthesis was induced by addition of L-arabinose (final concentration 0.2%). Samples for quantitative Western-blot analysis were withdrawn 30 min after *reaL* induction. The strains are indicated on top. The position of RpoS and ribosomal protein S1 (loading control) are indicated at the right. **(B)** The strains PAO1Δ*reaL*(pKH6; pME6014-RpoS), PAO1Δ*reaL*(pKH6-ReaL; pME6014-RpoS), and PAO1Δ*reaL*(pKH6-ReaL_Δ11-18_; pME6014-RpoS) were grown in LB medium. At an OD_600_ of 2.5, ReaL synthesis was induced by addition of L-arabinose (final concentration 0.2%). Samples for β-galactosidase activity measurements were withdrawn 30 min after ReaL induction. The values shown were derived from three independent experiments. The error bars represent standard deviations.

As the modified GRIL-seq approach supported the hypothesis that ReaL inhibits *rpoS* translation, we next assessed the steady-state levels of RpoS protein in strains PAO1Δ*rpoS*, PAO1Δ*reaL*(pKH6), and PAO1Δ*reaL*(pKH6-ReaL). These strains were cultured in LB until they reached an OD_600_ of 1. Then, ReaL synthesis was induced with L-arabinose, and the cells were further grown for 30 min. The RpoS levels were assessed through quantitative Western-blotting. A lower OD_600_ value for ReaL induction was chosen for this analysis to minimize detection of RpoS synthesized prior to ReaL induction. In contrast to strain PAO1Δ*reaL*(pKH6), the RpoS levels were greatly diminished (>10-fold) in the ReaL over-producing strain PAO1Δ*reaL*(pKH6-ReaL) (Figure 3A).

To verify this finding, a translational *rpoS::lacZ* reporter gene, mounted on plasmid pME6014-RpoS, was employed. Strain PAO1Δ*reaL*(pME6014-RpoS) was transformed with plasmids pKH6, pKH6-ReaL, and pKH6-ReaL_Δ11-18_, respectively. Plasmid pKH6-ReaL_Δ11-18_ encodes a truncated variant of ReaL lacking nucleotides 11 - 18, which are predicted to interact with *rpoS* mRNA (Figure 2A). The β-galactosidase activities conferred by plasmid pME6014-RpoS in strains PAO1Δ*reaL*(pKH6; pME6014-RpoS) and PAO1Δ*reaL*(pKH6-ReaL; pME6014-RpoS) (Figure 3B) showed that *reaL* expression leads to a significant reduction of *rpoS::lacZ* translation. However, expression of the *reaL*_Δ11-18_ variant resulted in β-galactosidase activities similar to those observed with the control strain PAO1Δ*reaL*(pKH6; pME6014-RpoS). As the ReaL levels were similar in strains PAO1Δ*reaL*(pKH6-ReaL; pME6014-RpoS), and PAO1Δ*reaL*(pKH6-ReaL_Δ11-18;_ pME6014-RpoS) (Supplementary Figure S2), and as ReaL synthesis did not impact on the expression of a transcriptional *rpoS-lacZ* fusion gene (Supplementary Figure S4), we concluded that the 5′-terminal sequence of ReaL is required for base-pairing with and to inhibit *rpoS* translation.

The study by Carloni et al. (2017) indicated that ReaL acts by base-pairing with *pqsC* mRNA and that it positively stimulates translation of the *pqsC* gene in a Hfq-independent manner. To test whether ReaL-mediated translational silencing of *rpoS* mRNA requires Hfq, we compared the β-galactosidase activities conferred by plasmid pME6014-*rpoS* in strains PAO1 (pKH6-ReaL; pME6014-RpoS), PAO1(pKH6; pME6014-RpoS), PAO1Δ*hfq*(pKH6-ReaL; pME6014-RpoS), and PAO1Δ*hfq*(pKH6; pME6014-RpoS). In contrast to strain PAO1(pKH6-ReaL; pME6014-RpoS), *rpoS* translation did not decrease in the *hfq* deletion strain (Supplementary Figure S5), suggesting that negative regulation of *rpoS* by ReaL is Hfq-dependent.

## Discussion

Here, we identified ReaL as the first known bacterial sRNA, which apparently represses *rpoS* translation by a base-pairing mechanism in a Hfq-dependent manner. The interaction between ReaL and *rpoS* mRNA is supported by the modified GRIL-seq approach (Figure 2B) and by the observation that ReaL_D11-18_ was defective in repressing *rpoS* translation (Figure 3B). We attempted to obtain further evidence for the ReaL-*rpoS* mRNA interaction by introducing mutations into the TIR of *rpoS* mRNA. However, compensatory mutations in the 5′ UTR of *rpoS* could not be constructed because of their effect on translation initiation due to the disruption of the Shine-Dalgarno sequence; indeed, all attempted mutations affected translation of *rpoS* mRNA *per se*. This excluded the possibility to study ReaL-mediated regulation of *rpoS* in more detail. In any case, ReaL overproduction reduced the *rpoS* transcript (Supplementary Table S3 and Figure S3) and protein levels (Figure 3A), respectively. It turn, translational silencing of *rpoS* mRNA resulted in faster degradation of the mRNA in the presence of ReaL (Supplementary Figure S3) as previously observed for RyhB-mediated translational repression of *sodB* mRNA (Massé and Gottesman, 2002; Afonyushkin et al., 2005).

**FIGURE 4:**
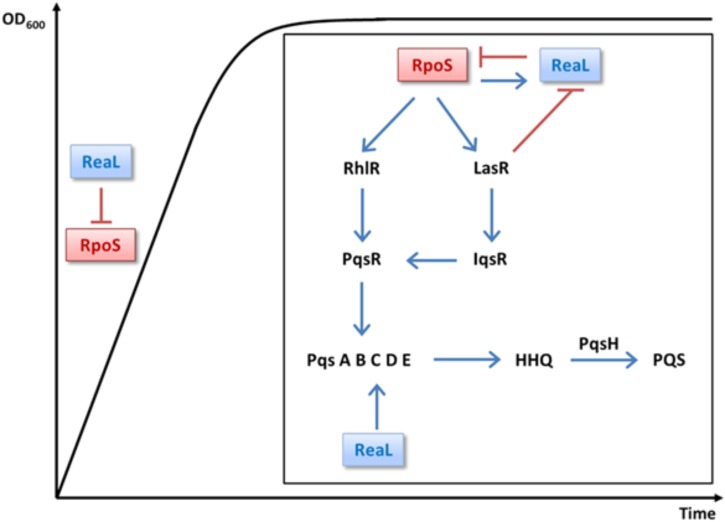
Integration of ReaL in the multi-layered quorum sensing cascade. σ^70^-dependent *reaL* transcription occurs during logarithmic growth (Sorger-Domenigg, 2010; Carloni et al., 2017). Continuous ReaL synthesis may prevent *rpoS* translation during logarithmic growth to avoid needless gene expression. When the cells encounter stationary phase, RpoS-mediated transcription of *reaL* increases through RpoS (Carloni et al., 2017), which in turn leads to repression of *rpoS* translation by a mixed negative feedback loop. When the cell density increases, LasR bound to its cognate autoinducer represses *reaL* transcription (Carloni et al., 2017), alleviating ReaL-mediated repression of RpoS synthesis, which in turn would stimulate LasR and RhlR synthesis (Schuster et al., 2004), and consequently the synthesis of PqsR and IqsR. Thus, in conjunction with other upstream regulators (Lee and Zhang, 2015), RpoS would act on top of a coherent feed-forward loop that serves to induce QS dependent genes. Positive and negative regulation is denoted by arrows and bars, respectively.

By comparing the transcriptomes of PAO1 and a PAO1D*rpoS* strain, Schuster et al. (2004) reported that RpoS positively regulates the QS response regulator genes *lasR* and *rhlR* ∼2-fold, which is in agreement with our RNA_Seq_ analysis performed with the strains PAO1Δ*reaL*(pKH6) and PAO1Δ*reaL*(pKH6-ReaL) (not shown). Hence, several differentially abundant transcripts/operons (Figure 1) and QS-mediated phenotypes reported upon over-production of ReaL (Carloni et al., 2017) might result from RpoS-mediated regulation of *lasR*/*rhlR*. When QS comes into effect the LasR regulator is the first to be activated, which in turn activates the Rhl, Pqs, and Iqs systems (Lee and Zhang, 2015). Thus, ReaL-mediated repression of *rpoS*, and consequently reduced synthesis of *lasR* would explain the diminished transcription of LasR-dependent genes/operons, such as the *psl* operon (Supplementary Table S3; Gilbert et al., 2009), encoding functions for matrix polysaccharide- or functions required for pyoverdine synthesis (Supplementary Table S3). The increase in pyocyanin production observed after ReaL overproduction (Carloni et al., 2017) is phenotypically in accord with the increased production of pyocyanin observed in a PAO1D*rpoS* strain (Suh et al., 1999; Whiteley et al., 2000; Diggle et al., 2002). However, ReaL-mediated over-production of pyocyanin was LasR/I independent (Carloni et al., 2017), and can therefore not be reconciled with its positive impact on LasR transcription (Schuster et al., 2004). ReaL-mediated stimulation of pyocyanin production was dependent on RhlR (Carloni et al., 2017), which can regulate PQS production in a positive (Dekimpe and Deziel, 2009) as well as in a negative manner (Brouwer et al., 2014). Given the complexity and interdependence of QS regulation more studies are required to test how ReaL-mediated negative regulation of *rpoS* impacts on the Rhl system and how this affects pyocyanin production. The increase in pyocyanin synthesis upon ReaL overproduction has been mainly attributed to ReaL-mediated translational stimulation of the *pqsC* gene, the function of which is required for production of the quinolone signal PQS (Figure 4; Carloni et al., 2017). However, as ReaL regulates several QS-dependent genes (Supplementary Table S3), which are likewise regulated by RpoS, its impact seems not to be restricted to PQS synthesis but it seems to operate as well in a hierarchical manner by acting on top of the cascade (Figure 4).

Despite the similarities in phenotypes and in the transcriptional profiles observed for the *rpoS* deletion mutant (Schuster et al., 2004) and the ReaL over-producing strain, a large number of genes displayed differential abundance in either strains (Supplementary Table S3). On one hand, these differences may arise from the different methods used for the transcriptome analysis, *viz* DNA microarrays (Schuster et al., 2004) and RNA_Seq_ (Supplementary Table S3). On the other hand, it is possible that the regulatory role of ReaL extends beyond *rpoS* repression.

A working model, which integrates RpoS and ReaL in the multi-layered QS network is presented in Figure 4. s^70^-dependent *reaL* transcription is observed during logarithmic growth and peaks in stationary phase (Sorger-Domenigg, 2010; Carloni et al., 2017). Therefore, continuous ReaL synthesis may prevent *rpoS* translation during logarithmic growth to avoid superfluous gene expression. When the cells enter stationary phase, RpoS-mediated transcription of *reaL* increases through stimulation by RpoS, which in turn leads to repression of *rpoS* translation by a mixed negative feedback loop, as observed for RpoE and the sRNA RybB in *E. coli* (Thompson et al., 2007). When the cell density increases, LasR bound to its cognate autoinducer represses *reaL* transcription (Carloni et al., 2017), alleviating ReaL-mediated repression of RpoS synthesis, which in turn would stimulate LasR and RhlR synthesis (Schuster et al., 2004), and consequently the synthesis of PqsR and IqsR. Thus, in conjunction with other upstream regulators (Lee and Zhang, 2015), RpoS would act on top of a coherent feed-forward loop that serves to induce QS dependent genes.

## Author Contributions

UB and ES conceived and designed the experiments. HTBN, DRA, MT, and TS-D performed the experiments. UB, ES, HTBN, FA, and DRA analyzed the data. UB and DRA wrote the paper.

## Conflict of Interest Statement

The authors declare that the research was conducted in the absence of any commercial or financial relationships that could be construed as a potential conflict of interest.

## Footnotes

1 https://www.vbcf.ac.at/home/

2 http://www.kegg.jp/kegg-bin/show_organism?org=pau
