# Supplementary material for: Negative Control of RpoS Synthesis by the sRNA ReaL in Pseudomonas aeruginosa
Source: Front Microbiol. 2018 Oct 29;9:2488. doi: 10.3389/fmicb.2018.02488 (PMC6215814; doi:10.3389/fmicb.2018.02488)
Supplement: Supplementary file 1 [file Data_Sheet_1.docx]

Supplementary Materials

Negative control of RpoS synthesis by the sRNA ReaL in *Pseudomonas aeruginosa*

Hue Thi Bach Nguyen^1^*, David Romero A. ^1^*, Fabian Amman^2^, Theresa Sorger-Domenigg^1^, Muralidhar Tata^1^, Elisabeth Sonnleitner^1^ and Udo Bläsi^1#^

^#^ **Correspondence:** Corresponding Author: Udo.Blaesi@univie.ac.at

## SUPPLEMENTARY MATERIALS

**Supplementary Figure S1** Genetic organization, expression signals and expression of *real* in Pae strain PAO1. **(A)** *reaL* (black arrow) is located between the open reading frames PA3535 and PA3536 (grey arrows). **(B)** Total RNA of PAO1 grown in LB medium to an OD_600_ of 2,0 was isolated, and ReaL was detected by Northern-blotting using the strand-specific probe W28 (Supplementary Table S2). The sizes (in nucleotides) of RNA markers (M) are indicated at the left. **(C)** The sequence of *reaL* is shown in bold. The σ^70^ promoter (-35 and -10 signatures) is underlined and in italics, and the RpoS consensus motif 5´-CTATACT-3´ (Schuster et al., 2004), overlapping with the -10 region of the σ^70^ promoter is highlighted in blue. The 5´ and 3´ ends mapped by 5´-3´ circularization are indicated by arrows. The transcriptional start site is shown underlined and in italics. The stop codon of PA3535 is underlined. The complementary sequence of primer W28 is underlined and in bold. The sequence of oligonucleotide N29 (Supplementary Table S2) is boxed.

**Supplementary Figure S2** Levels of ReaL and ReaL_Δ11-18_ in strains PAO1Δ*reaL*(pKH6), PAO1Δ*reaL*(pKH6-ReaL) and PAO1Δ*reaL*(pKH6-ReaL_Δ11-18_). The strains were grown to an OD_600_ of 2.5 in LB broth. Then, synthesis of ReaL and the ReaL_Δ11-18_ variant was induced with L-arabinose (0.2% final concentration) and the cells were cultured for another 30 min. Total RNA was isolated and ReaL was detected by Northern-blotting using the strand-specific probe W28 (Supplementary Table S2). 5S rRNA was used as a loading control. The strains are indicated on top. ReaL and 5S rRNA are indicated at the right. Only the relevant parts of the autoradiogram are shown.

**Supplementary Figure S3** Determination of the steady state levels and half-life of *rpoS* mRNA by RT-qPCR in the presence and absence of ReaL. Strains PAO1Δ*reaL*(pKH6) and PAO1Δ*reaL*(pKH6-ReaL) were grown in LB medium supplemented with 50 µg/ml gentamycin until they reached an OD_600_ of 2.5. Then ReaL synthesis was induced with L-arabinose (final concentration 0.2 %). After 30 min (t_0_) the first samples -revealing the steady state levels of *rpoS* mRNA normalized to *rpoD* mRNA levels in the presence and absence of ReaL- were withdrawn. The *rpoS* mRNA levels at t_0_ were ~18-fold reduced in PAO1Δ*reaL*(pKH6-ReaL) when compared with PAO1Δ*reaL*(pKH6). After withdrawal of the first samples rifampicin was added. Further samples were then withdrawn after 1 (t_1_), 2 (t_2_), 5 (t_3_), and 7.5 (t_4_) min, respectively. The half-life was ~5-fold reduced in PAO1Δ*reaL*(pKH6-ReaL) when compared with PAO1Δ*reaL*(pKH6). The copy number of *rpoS* mRNA (y-axis) at the different time points was determined as described in Materials and Methods. The results represent the average of two independent experiments including each three technical replicates. The values represent the means and SDs (standard deviations) of changes in *rpoS* levels. The error bars represent SDs.

**Supplementary Figure S4** ReaL does not affect *rpoS* transcription. The strains PAO1Δ*reaL*(pKH6; pME6016-RpoS) and PAO1Δ*reaL*(pKH6-ReaL; pME6016-RpoS) were grown aerobically at 37^o^C in LB broth. At an OD_600_ of 2.5, the synthesis of ReaL was induced with L-arabinose (0.2% final concentration) and the cells were cultured for another 30 min. Then, the β-galactosidase activities were determined for the different strains. The bars depict the β-galactosidase values conferred by the transcriptional *rpoS-lacZ* fusion encoded by plasmid pME6016-RpoS in strains PAO∆*real*(pKH6) and PAO1∆*real*(pKH6-ReaL). The error bars represent standard deviations from three independent experiments.

**Supplementary Figure S5** ReaL-mediated repression of *rpoS* translation is Hfq-dependent. The strains PAO1(pKH6-ReaL; pME6014-RpoS), PAO1(pKH6; pME6014-RpoS), PAO1Δ*hfq*(pKH6-ReaL; pME6014-RpoS) and PAO1Δ*hfq*(pKH6; pME6014-RpoS) were grown aerobically at 37^o^C in LB broth. At an OD_600_ of 2.5, the synthesis of ReaL was induced by addition of L-arabinose (0.2% final concentration). After 30 min, the β-galactosidase activities were determined for the different strains. The bars depict the β-galactosidase values conferred by the translational *rpoS::lacZ* fusion in the indicated strains. The error bars represent standard deviations from three independent experiments.

**Supplementary Table S1** Strains and plasmids used in this study.

**Supplementary Table S2** Oligonucleotides used in this study. Restriction sites are indicated in bold. The binding regions correspond to the genome coordinates of PAO1 (AE004091; http://www.pseudomonas.com).

**Supplementary Table S3** Differential abundance of transcripts in strains PAO1Δ*reaL*(pKH6-ReaL) and PAO1Δ*reaL*(pKH6) revealed by RNA_Seq_ and strains PAO1Δ*rpoS* and PAO1 revealed with DNA microarrays (Schuster et al., 2004), respectively. For the RNA_Seq_ analyses only transcripts that showed a log_2_ ± 1.5 -fold change and a p-value ≤ 0.05 were considered to be significant. Gene numbers and functions are taken from the Pseudomonas genome database (http://www.pseudomonas.com).

**Supplementary Figure S1**

**
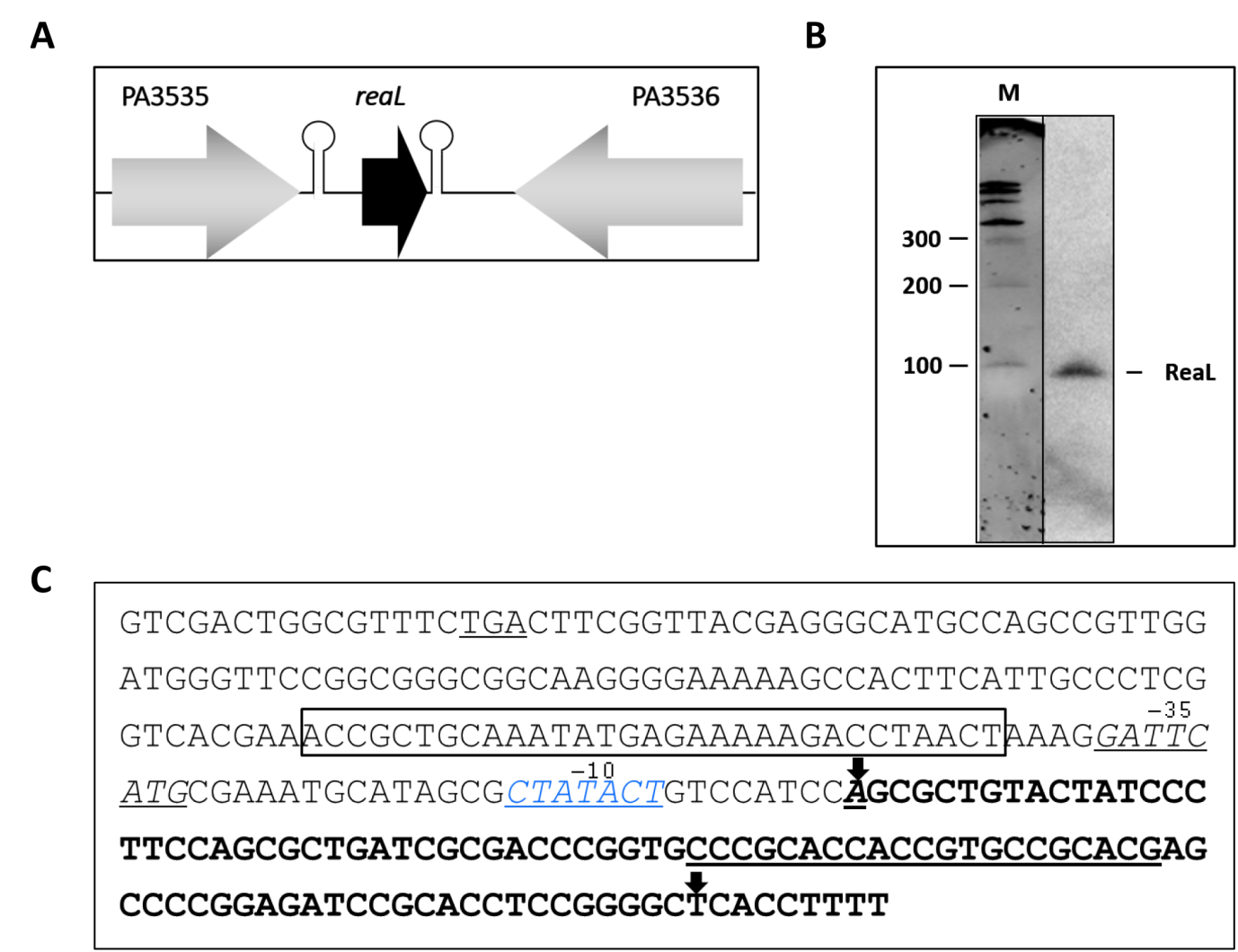
**

**Supplementary Figure S2**

**
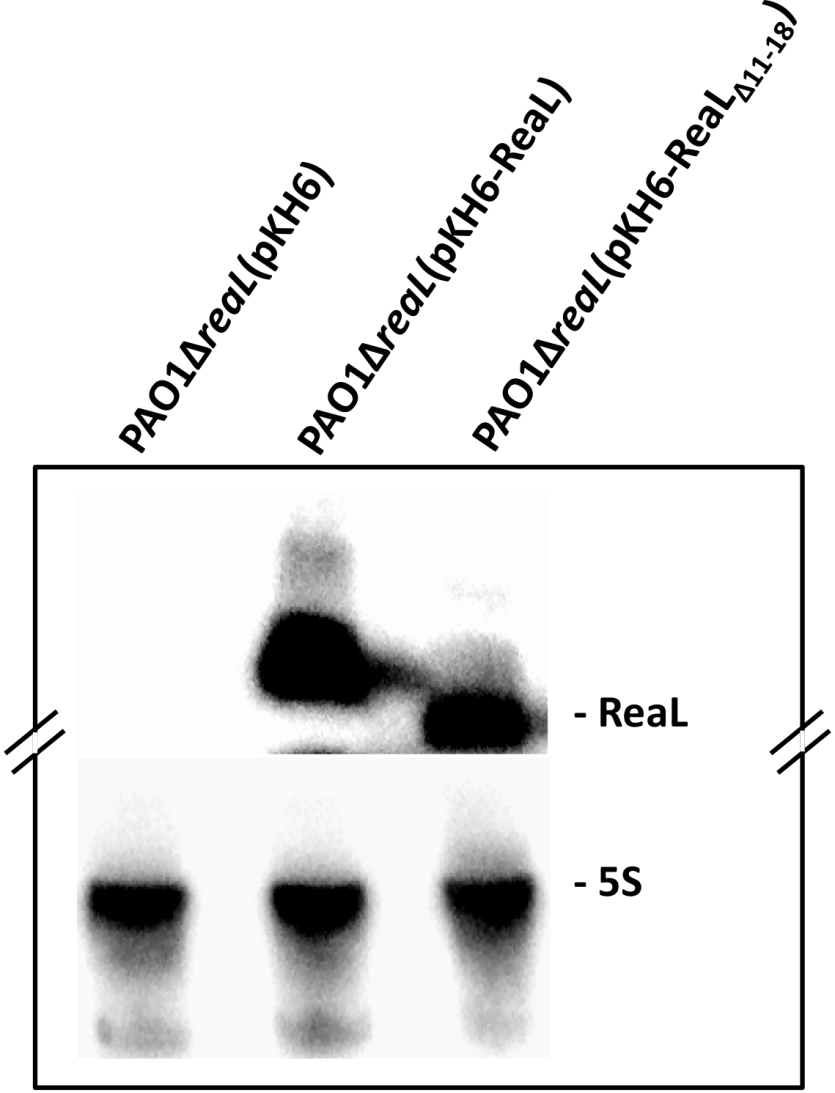
**

**Supplementary Figure S3**

**
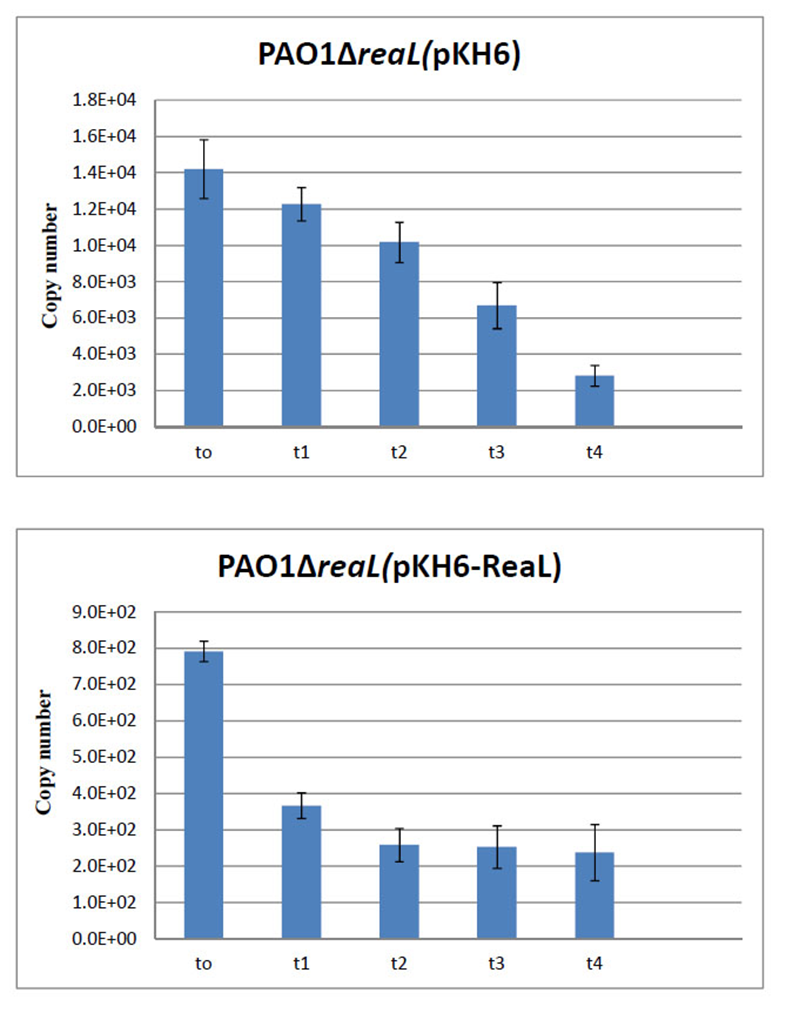
**

**Supplementary Figure S4**

**
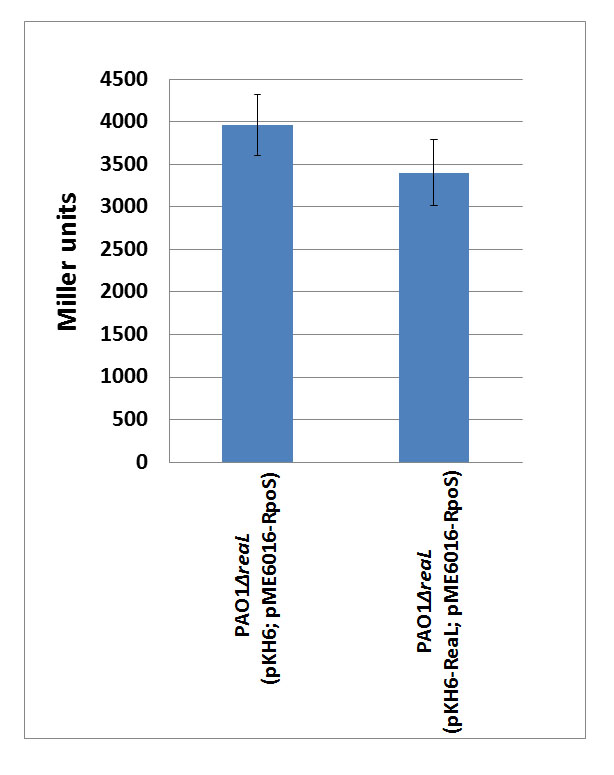
**

**Supplementary Figure S5**

**
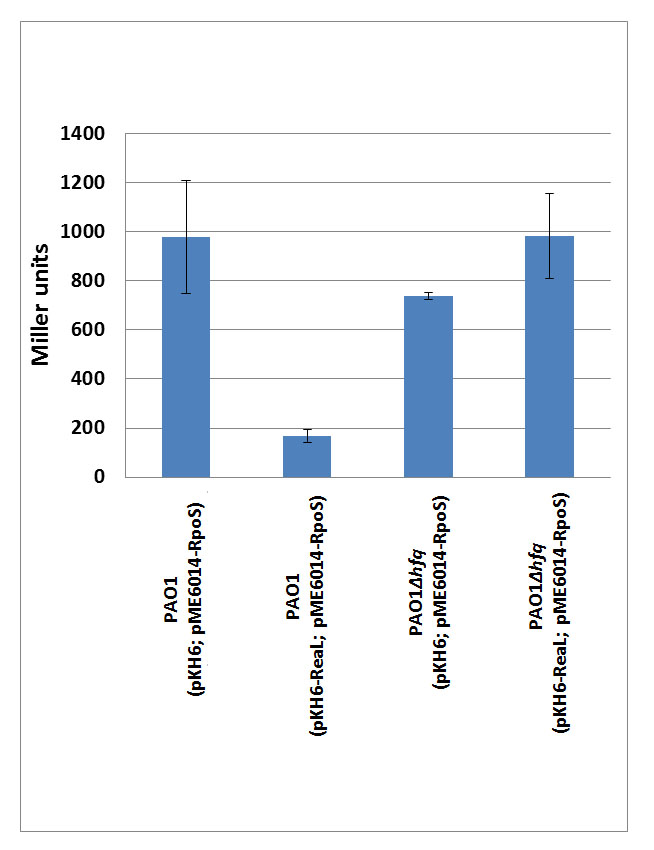
**

## Supplementary Tables

**Supplementary Table S1**

| **Strains/plasmid** | **Genotype/relevant features** | **References** |
| --- | --- | --- |
| ***Escherichia coli*** | | |
| S17-1 | *recA1 pro thi*; the *tra* genes of plasmid RP4 are integrated in the chromosome | (Simon et al., 1986) |
| ***Pseudomonas aeruginosa*** | | |
| PAO1 | Wild-type | (Holloway et al., 1979) |
| PAO1Δ*reaL* | In frame *reaL* deletion | This study |
| PAO1Δ*rpoS* | In frame *rpoS* deletion | (Suh et al., 1999) |
| PAO1Δ*hfq* | In frame *hfq* deletion | (Sonnleitner et al., 2017) |
| **Plasmids** | | |
| pKH6 | pJN105-derivated vector: expression of small RNA with TTS +1 | (Han et al., 2016) |
| pKH6-ReaL | pkH6 harboring *reaL* | This study |
| pKH6-ReaL_Δ11-18_ | pkH6 harboring *reaL* with a deletion spanning nt 11-18 | This study |
| pKH13-t4rnl1 | Plasmid encoding the T4RNA ligase gene | (Han et al., 2016) |
| pME6014 | Cloning vector for translational *lacZ* fusions, Tc^R^ | (Heeb et al., 2000) |
| pME6014-RpoS | In frame translational fusion of the first 56 codons of *rpoS* to *lacZ* | This study |
| pME6016 | Cloning vectors for transcriptional *lacZ* fusions, TcR | (Schnider-Keel et al., 2000) |
| pME6016-RpoS | Transcriptional in frame fusion of the first 56 codons of *rpoS* to *lacZ* | This study |
| pSUP202 | pBR325, Ap^R^, Cm^R^, Tc^R^, *mob* | (Simon et al., 1983) |
| pSUP202-ReaLup | pSUP202 harboring the *reaL* upstream region | This study |
| pSUP202-ReaLko | pSUP202-ReaLup harboring the *reaL* downstream region | This study |
| pUC19 | ColE1 replicon; Ap^R^ | (Norrander et al., 1983) |

**Supplementary Table S2**

| **Name** | **Sequence** | **Binding region** |
| --- | --- | --- |
| A52 | AAAA**GGATCC**GTTCCACCCGCAGTTAGTACG | 4059497-4059477 |
| B52 | TTTT**CTGCAG**GCGTGTAGTCGATGTGCTTGTG | 4058742-4058763 |
| B53 | GGCACCGGGTCGCGATCAG | 3958095-3958077 |
| C53 | CGCACGAGCCCCGGAGATC | 3958109-3958127 |
| E163 | TACGT**TCTAGA**GAGCGCTGTAC/CCAGCGCTGATCGCGACCC | 3958053-3958063  3958071-3958090 |
| I26 | CCCCACACTACCATCGGCGATGCGTCG | 6039619-6039645 |
| J98 | TCAAGGAGCTCAACGTCTAC | 4058384-4058365 |
| K98 | AGAGACGTCTACCGAAGTCA | 4058224- 4058243 |
| M13 | GTAAAACGACGGCCAG | Universal sequencing primer (ThermoFisher) |
| N29 | GC**TCTAGA**CCGCTGCAAATATGAGAAAAAGACCTAACT | 3957982-3958011 |
| Q32 | AAAAAA**CGATCG**ACGAGCCCCGGAGATCCGCAC | 3958112-3958132 |
| Q117 | AAGGCCCTGAAGAAGCACGG | 635516- 635497 |
| R32 | AAAAA**GAATTC**CACACGGGAGAAACACTCGC | 3958716-3958697 |
| R117 | GATCGGCATGAACAGCTCGG | 635436- 635455 |
| V34 | GGCACCGGGTCGCGATCAG | 3958095-3958077 |
| W28 | CGTGCGGCACGGTGGTGCGGG | 3958114-3958094 |
| W34 | CGCACGAGCCCCGGAGATC | 3958109-3958127 |
| Y138 | TACGT**TCTAGA**GAGCGCTGTACTATCCCTTCCAG | 3958053-3958074 |
| Y144 | AGCGCTGTACTATCCCTT | 3958053-3958070 |
| Z138 | TACGT**CTGCAG**AAAAGGTGAGCCCCGGAGGTGCG | 3958150-3958128 |
| Z144 | CGCGCGCGTGTAGTCGATG | 4058737-4058755 |

**Supplementary Table S3**

| Gene | This study | | Schuster et al. (2004) | Description |
| --- | --- | --- | --- | --- |
|  | p-value | Fold-change (log_2_) PAO1Δ*reaL* (pKH6-ReaL) vs PAO1Δ*reaL* (pKH6) | Fold change PAO1Δ*rpoS* vs PAO1 |  |
| PA0024_hemF | 1.9E-15 | 1.64 |  | coproporphyrinogen III oxidase, aerobic |
| PA0045 | 2.8E-29 | 2.31 | 2.2 | Hypothetical, unclassified, unknown |
| PA0046 | 3.4E-29 | 2.02 |  | Hypothetical, unclassified, unknown |
| PA0047 | 4.1E-33 | 2.06 |  | Hypothetical, unclassified, unknown |
| PA0052 | 2.9E-31 | -1.96 | -7.1 | Hypothetical, unclassified, unknown |
| PA0059_osmC | 2.1E-31 | -1.95 |  | Adaptation, Protection |
| PA0060 | 1.4E-19 | -1.69 |  | Hypothetical, unclassified, unknown |
| PA0074_ppkA | 1.3E-87 | 2.71 |  | Adaptation, Protection; Translation, post-translational modification, degradation; Protein secretion/export apparatus |
| PA0075_pppA | 1.9E-36 | 2.35 |  | Putative enzymes; Protein secretion/export apparatus |
| PA0077_icmF1 | 3.0E-50 | 2.33 |  | Protein secretion/export apparatus |
| PA0085_hcp1 | 3.9E-72 | 2.83 | 2.7 | Secreted Factors (toxins, enzymes, alginate) |
| PA0090_clpV1 | 5.7E-79 | 2.54 | 2.8 | Translation, post-translational modification, degradation; Chaperones & heat shock proteins; Protein secretion/export apparatus |
| PA0091_vgrG1 | 2.4E-75 | 2.90 | 2.5 | Protein secretion/export apparatus |
| PA0103 | 9.1E-103 | -3.11 |  | Membrane proteins; Transport of small molecules |
| PA0104 | 1.4E-24 | -1.99 |  | Hypothetical, unclassified, unknown |
| PA0105_coxB | 2.2E-34 | -1.92 | -18.3 | Energy metabolism |
| PA0106_coxA | 4.9E-82 | -2.73 | -21.4 | Energy metabolism |
| PA0107 | 5.5E-93 | -2.73 | -12.1 | Energy metabolism |
| PA0108_coIII | 7.9E-81 | -2.63 | -9 | Energy metabolism |
| PA0110 | 4.3E-93 | -2.75 | -3.1 | Hypothetical, unclassified, unknown |
| PA0111 | 2.4E-52 | -2.59 | -4.7 | Hypothetical, unclassified, unknown |
| PA0112 | 7.1E-55 | -2.45 | -2.8 | Membrane proteins |
| PA0113 | 4.2E-56 | -2.38 | -2.8 | Energy metabolism |
| PA0126 | 2.9E-37 | 2.30 | 2.1 | Hypothetical, unclassified, unknown |
| PA0140_ahpF | 2.1E-08 | 2.51 |  | Adaptation, Protection |
| PA0175 | 1.4E-38 | -1.66 | -15.9 | Transcriptional regulators; Adaptation, Protection; Chemotaxis |
| PA0180_cttP | 2.3E-40 | -1.63 | -5.5 | Adaptation, Protection; Chemotaxis |
| PA0200 | 6.3E-23 | 1.64 |  | Hypothetical, unclassified, unknown |
| PA0261 | 9.9E-82 | 3.03 | 4.5 | Hypothetical, unclassified, unknown |
| PA0263_hcpC | 1.3E-77 | 6.18 | 3.3 | Secreted Factors (toxins, enzymes, alginate) |
| PA0276 | 2.9E-32 | 1.67 |  | Membrane proteins |
| PA0277 | 3.9E-15 | 1.53 | 2.4 | Hypothetical, unclassified, unknown |
| PA0293_aguB | 3.8E-22 | 1.84 |  | Amino acid biosynthesis and metabolism; Putative enzymes |
| PA0315 | 6.0E-21 | -1.80 | -4.1 | Hypothetical, unclassified, unknown |
| PA0355_pfpI | 4.3E-45 | -1.94 |  | Translation, post-translational modification, degradation; Antibiotic resistance and susceptibility; Motility & Attachment; Cell wall / LPS / capsule |
| PA0364 | 4.0E-39 | -1.95 |  | Putative enzymes |
| PA0365 | 6.2E-33 | -1.95 |  | Membrane proteins |
| PA0459 | 9.5E-66 | -2.50 | -18.3 | Translation, post-translational modification, degradation |
| PA0483 | 1.3E-20 | -1.52 | -5.2 | Putative enzymes |
| PA0484 | 1.1E-40 | -1.72 | -10.3 | Hypothetical, unclassified, unknown |
| PA0509_nirN | 1.5E-22 | 2.10 |  | Biosynthesis of cofactors, prosthetic groups and carriers; Energy metabolism |
| PA0511_nirJ | 9.1E-20 | 1.91 |  | Biosynthesis of cofactors, prosthetic groups and carriers; Energy metabolism |
| PA0519_nirS | 3.6E-05 | 1.75 |  | Energy metabolism |
| PA0522 | 3.4E-18 | 1.81 |  | Hypothetical, unclassified, unknown |
| PA0523_norC | 1.9E-27 | 2.15 |  | Energy metabolism |
| PA0524_norB | 6.4E-26 | 2.17 |  | Energy metabolism |
| PA0525 | 6.1E-17 | 1.87 |  | Energy metabolism |
| PA0540 | 3.1E-40 | -2.47 | -3.2 | Hypothetical, unclassified, unknown |
| PA0563 | 3.5E-23 | 1.65 |  | Hypothetical, unclassified, unknown |
| PA0565 | 8.3E-23 | -1.57 | -3.8 | Hypothetical, unclassified, unknown |
| PA0585 | 1.2E-22 | -1.50 | -2.1 | Hypothetical, unclassified, unknown |
| PA0586 | 5.3E-53 | -1.61 | -6.2 | Hypothetical, unclassified, unknown |
| PA0587 | 8.7E-48 | -1.56 | -6.3 | Hypothetical, unclassified, unknown |
| PA0588 | 1.7E-50 | -1.57 | -4.7 | Hypothetical, unclassified, unknown |
| PA0672_hemO | 3.1E-05 | -1.70 |  | Biosynthesis of cofactors, prosthetic groups and carriers |
| PA0704 | 6.6E-62 | -1.97 | -24.2 | Putative enzymes |
| PA0776 | 2.2E-18 | -1.55 | -11.9 | Hypothetical, unclassified, unknown |
| PA0798_pmtA | 2.5E-68 | -2.67 | -6.2 | Fatty acid and phospholipid metabolism |
| PA0830 | 4.6E-83 | 2.39 |  | Hypothetical, unclassified, unknown |
| PA0849_trxB2 | 6.6E-26 | 2.49 |  | Nucleotide biosynthesis and metabolism |
| PA0959 | 3.1E-29 | -1.62 | -3.2 | Hypothetical, unclassified, unknown |
| PA0960 | 2.9E-19 | -1.63 | -2.8 | Hypothetical, unclassified, unknown |
| PA1042 | 4.0E-117 | -2.77 | -2.5 | Membrane proteins |
| PA1043 | 2.5E-43 | -1.68 |  | Hypothetical, unclassified, unknown |
| PA1066 | 2.6E-32 | -1.64 | -4.3 | Putative enzymes |
| PA1112 | 4.7E-29 | -1.55 | -2 | Hypothetical, unclassified, unknown |
| PA1121_yfiR | 2.6E-37 | -1.68 | -4.9 | Cell wall / LPS / capsule |
| PA1166 | 1.6E-55 | -2.01 | -13.6 | Hypothetical, unclassified, unknown |
| PA1175_napD | 4.4E-20 | -1.52 | -16.5 | Energy metabolism |
| PA1176_napF | 3.9E-75 | -1.88 | -39 | Energy metabolism |
| PA1190 | 3.5E-29 | -1.81 | -3.3 | Membrane proteins |
| PA1289 | 6.1E-61 | -2.30 | -30.6 | Hypothetical, unclassified, unknown |
| PA1300 | 4.4E-17 | -1.91 |  | Transcriptional regulators |
| PA1301 | 1.2E-14 | -2.02 |  | Membrane proteins; Transcriptional regulators |
| PA1316 | 8.5E-26 | -1.84 |  | Membrane proteins; Transport of small molecules |
| PA1327 | 1.8E-49 | -1.89 | -4.8 | Putative enzymes |
| PA1344 | 1.8E-34 | -1.64 | -7 | Putative enzymes |
| PA1345 | 2.9E-45 | -2.08 | -3.3 | Hypothetical, unclassified, unknown |
| PA1349 | 2.1E-27 | -1.99 | -3.1 | Hypothetical, unclassified, unknown |
| PA1351 | 1.4E-49 | -2.01 | -2.2 | Transcriptional regulators |
| PA1353 | 7.5E-64 | -2.54 | -3.6 | Hypothetical, unclassified, unknown |
| PA1354 | 3.7E-59 | -2.49 | -6.3 | Hypothetical, unclassified, unknown |
| PA1355 | 2.5E-34 | -2.56 | -9 | Hypothetical, unclassified, unknown |
| PA1356 | 2.3E-88 | -2.92 | -8.1 | Hypothetical, unclassified, unknown |
| PA1358 | 1.7E-35 | -1.89 | -3.8 | Hypothetical, unclassified, unknown |
| PA1366 | 1.4E-13 | -1.51 | -2.7 | Hypothetical, unclassified, unknown |
| PA1403 | 5.9E-20 | -1.56 |  | Transcriptional regulators |
| PA1407 | 5.9E-33 | -1.61 |  | Hypothetical, unclassified, unknown |
| PA1408 | 6.4E-41 | -1.67 |  | Membrane proteins |
| PA1471 | 2.6E-24 | -1.71 |  | Hypothetical, unclassified, unknown |
| PA1493_cysP | 2.1E-86 | 2.40 |  | Transport of small molecules |
| PA1512_hcpA | 3.4E-07 | 2.63 |  | Secreted Factors (toxins, enzymes, alginate) |
| PA1522 | 2.4E-32 | -1.53 |  | Hypothetical, unclassified, unknown |
| PA1523_xdhB | 4.1E-26 | -1.52 | -1.8 | Nucleotide biosynthesis and metabolism |
| PA1540 | 4.9E-12 | -1.96 |  | Membrane proteins |
| PA1606 | 1.8E-20 | -1.69 |  | Hypothetical, unclassified, unknown |
| PA1621 | 1.6E-46 | 1.68 |  | Putative enzymes |
| PA1622 | 5.4E-47 | 1.84 |  | Putative enzymes |
| PA1670_stp1 | 2.9E-248 | 3.98 | 3.8 | Translation, post-translational modification, degradation; Protein secretion/export apparatus |
| PA1671_stk1 | 7.5E-262 | 4.58 | 4 | Translation, post-translational modification, degradation; Protein secretion/export apparatus |
| PA1690_pscU | 9.0E-24 | 1.54 |  | Protein secretion/export apparatus |
| PA1691_pscT | 4.2E-15 | 1.63 |  | Protein secretion/export apparatus |
| PA1692 | 3.2E-20 | 1.51 |  | Protein secretion/export apparatus |
| PA1725_pscL | 1.2E-26 | 1.56 |  | Protein secretion/export apparatus |
| PA1728 | 9.1E-58 | -2.18 | -4.3 | Hypothetical, unclassified, unknown |
| PA1745 | 9.1E-20 | -1.59 | -6.6 | Hypothetical, unclassified, unknown |
| PA1777_oprF | 2.1E-31 | -1.64 |  | Membrane proteins; Transport of small molecules |
| PA1784 | 1.1E-92 | -2.66 | -16.8 | Hypothetical, unclassified, unknown |
| PA1806_fabI | 7.8E-52 | 1.76 | 1.8 | Fatty acid and phospholipid metabolism |
| PA1860 | 1.5E-66 | -2.37 | -5.6 | Putative enzymes |
| PA1867_xphA | 1.1E-04 | 1.65 |  | Protein secretion/export apparatus |
| PA1871_lasA | 1.3E-25 | -1.61 |  | Secreted Factors (toxins, enzymes, alginate); Translation, post-translational modification, degradation |
| PA1875 | 2.2E-62 | -2.59 | -61.3 | Protein secretion/export apparatus; Hypothetical, unclassified, unknown; Antibiotic resistance and susceptibility |
| PA1877 | 1.2E-43 | -1.86 | -11.5 | Protein secretion/export apparatus; Antibiotic resistance and susceptibility |
| PA1892 | 5.1E-24 | 1.65 |  | Hypothetical, unclassified, unknown |
| PA1913 | 1.1E-31 | 1.65 |  | Hypothetical, unclassified, unknown |
| PA1914 | 2.5E-41 | -2.41 | -111.4 | Putative enzymes |
| PA1921 | 8.2E-20 | -1.86 |  | Hypothetical, unclassified, unknown |
| PA1927_metE | 2.1E-41 | 1.56 |  | Amino acid biosynthesis and metabolism |
| PA1930 | 1.8E-51 | -1.98 | -16.3 | Adaptation, Protection; Chemotaxis |
| PA1931 | 2.3E-25 | -1.64 |  | Energy metabolism; Carbon compound catabolism |
| PA1942 | 2.2E-19 | 2.41 |  | Hypothetical, unclassified, unknown |
| PA1979_eraS | 1.9E-14 | -1.88 |  | Two-component regulatory systems |
| PA1980_eraR | 6.3E-10 | -1.61 |  | Transcriptional regulators; Two-component regulatory systems |
| PA1985_pqqA | 1.9E-03 | -1.64 |  | Biosynthesis of cofactors, prosthetic groups and carriers |
| PA1986_pqqB | 7.9E-32 | -1.53 |  | Biosynthesis of cofactors, prosthetic groups and carriers |
| PA1987_pqqC | 4.0E-26 | -1.53 |  | Biosynthesis of cofactors, prosthetic groups and carriers |
| PA1988_pqqD | 4.5E-23 | -1.51 |  | Biosynthesis of cofactors, prosthetic groups and carriers |
| PA2030 | 1.7E-22 | -1.71 |  | Hypothetical, unclassified, unknown |
| PA2033 | 1.3E-20 | -2.12 |  | Hypothetical, unclassified, unknown |
| PA2034 | 1.3E-13 | -1.96 |  | Hypothetical, unclassified, unknown |
| PA2038 | 1.5E-08 | 1.75 |  | Hypothetical, unclassified, unknown |
| PA2071_fusA2 | 5.6E-47 | -1.86 | -5.4 | Translation, post-translational modification, degradation |
| PA2072 | 1.7E-69 | -1.80 | -4.2 | Membrane proteins |
| PA2084 | 3.7E-26 | -1.57 |  | Amino acid biosynthesis and metabolism |
| PA2128_cupA1 | 3.3E-99 | 3.04 | 3.4 | Motility & Attachment |
| PA2129_cupA2 | 3.1E-39 | 2.93 |  | Motility & Attachment; Chaperones & heat shock proteins |
| PA2130_cupA3 | 3.5E-26 | 1.86 |  | Motility & Attachment |
| PA2131_cupA4 | 3.8E-29 | 2.62 |  | Motility & Attachment |
| PA2132_cupA5 | 2.5E-14 | 2.38 |  | Motility & Attachment; Chaperones & heat shock proteins |
| PA2133 | 1.2E-11 | 1.64 |  | Hypothetical, unclassified, unknown |
| PA2137 | 7.3E-28 | -2.09 |  | Hypothetical, unclassified, unknown |
| PA2138 | 1.5E-49 | -2.02 |  | DNA replication, recombination, modification and repair |
| PA2159 | 5.9E-19 | -1.82 |  | Hypothetical, unclassified, unknown |
| PA2166 | 1.2E-37 | -2.65 | -2 | Hypothetical, unclassified, unknown |
| PA2167 | 2.2E-29 | -1.73 |  | Hypothetical, unclassified, unknown |
| PA2168 | 1.0E-27 | -1.69 |  | Hypothetical, unclassified, unknown |
| PA2169 | 2.1E-19 | -1.74 |  | Hypothetical, unclassified, unknown |
| PA2170 | 5.6E-18 | -1.82 |  | Hypothetical, unclassified, unknown |
| PA2171 | 5.1E-16 | -1.54 |  | Hypothetical, unclassified, unknown |
| PA2172 | 2.2E-15 | -1.62 |  | Hypothetical, unclassified, unknown |
| PA2173 | 2.5E-16 | -1.72 |  | Hypothetical, unclassified, unknown |
| PA2174 | 1.6E-56 | -2.49 | -4.7 | Hypothetical, unclassified, unknown |
| PA2182 | 5.8E-25 | -1.62 | -3.3 | Hypothetical, unclassified, unknown |
| PA2183 | 4.4E-13 | -1.54 |  | Hypothetical, unclassified, unknown |
| PA2184 | 2.3E-22 | -1.72 |  | Hypothetical, unclassified, unknown |
| PA2186 | 1.3E-10 | -1.51 |  | Hypothetical, unclassified, unknown |
| PA2190 | 1.8E-31 | -2.16 |  | Hypothetical, unclassified, unknown |
| PA2204 | 1.5E-22 | 1.73 | 2 | Transport of small molecules |
| PA2207 | 9.1E-07 | -1.62 |  | Hypothetical, unclassified, unknown |
| PA2225 | 2.2E-32 | -1.80 | -2.8 | Hypothetical, unclassified, unknown |
| PA2228 | 3.5E-10 | -1.54 |  | Hypothetical, unclassified, unknown |
| PA2231_pslA | 1.1E-35 | -1.51 | -6.5 | Cell wall / LPS / capsule |
| PA2232_pslB | 2.4E-40 | -1.55 | -6.3 | Cell wall / LPS / capsule |
| PA2233_pslC | 2.2E-29 | -1.63 | -5.4 | Putative enzymes; Cell wall / LPS / capsule |
| PA2234_pslD | 3.0E-35 | -1.73 | -7 | Cell wall / LPS / capsule; Transport of small molecules |
| PA2235_pslE | 1.2E-38 | -1.65 | -7.9 | Cell wall / LPS / capsule |
| PA2236_pslF | 5.2E-35 | -1.70 | -7.2 | Cell wall / LPS / capsule |
| PA2245_pslO | 1.6E-16 | -1.69 |  | Hypothetical, unclassified, unknown |
| PA2268 | 1.9E-15 | -1.64 |  | Hypothetical, unclassified, unknown |
| PA2289 | 7.2E-35 | -1.79 | -3.6 | Hypothetical, unclassified, unknown |
| PA2290_gcd | 4.6E-66 | -2.09 | -5.1 | Carbon compound catabolism; Energy metabolism |
| PA2291 | 3.9E-22 | -1.62 |  | Transport of small molecules |
| PA2314 | 2.4E-15 | -1.75 |  | Membrane proteins; Transport of small molecules |
| PA2327 | 3.3E-41 | 1.58 | 1.7 | Membrane proteins; Transport of small molecules |
| PA2328 | 1.8E-52 | 1.67 |  | Hypothetical, unclassified, unknown |
| PA2363 | 4.5E-60 | -2.12 | -6.2 | Hypothetical, unclassified, unknown |
| PA2364 | 1.6E-35 | -1.61 | -4.9 | Hypothetical, unclassified, unknown |
| PA2375 | 6.4E-26 | -1.97 | -14.9 | Membrane proteins |
| PA2384 | 5.0E-34 | -2.55 |  | Hypothetical, unclassified, unknown |
| PA2385_pvdQ | 4.2E-07 | -2.30 |  | Adaptation, Protection |
| PA2386_pvdA | 3.1E-09 | -2.43 |  | Adaptation, Protection |
| PA2392_pvdP | 9.1E-14 | -1.96 |  | Adaptation, Protection |
| PA2393 | 7.4E-11 | -1.95 |  | Central intermediary metabolism |
| PA2394_pvdN | 3.8E-05 | -2.03 |  | Adaptation, Protection |
| PA2395_pvdO | 1.8E-10 | -2.11 |  | Adaptation, Protection |
| PA2397_pvdE | 1.3E-07 | -2.14 |  | Membrane proteins; Adaptation, Protection; Transport of small molecules |
| PA2399_pvdD | 1.5E-44 | -2.18 |  | Secreted Factors (toxins, enzymes, alginate); Adaptation, Protection |
| PA2400_pvdJ | 2.8E-28 | -2.15 |  | Adaptation, Protection |
| PA2402 | 1.2E-23 | -2.21 |  | Putative enzymes |
| PA2403 | 1.6E-28 | -2.08 |  | Membrane proteins |
| PA2404 | 2.1E-38 | -2.26 |  | Membrane proteins |
| PA2405 | 3.4E-33 | -2.36 |  | Hypothetical, unclassified, unknown |
| PA2406 | 3.0E-28 | -2.48 |  | Hypothetical, unclassified, unknown |
| PA2407 | 7.3E-18 | -1.69 |  | Motility & Attachment |
| PA2408 | 5.0E-14 | -1.75 |  | Transport of small molecules |
| PA2409 | 1.7E-10 | -1.55 |  | Membrane proteins; Transport of small molecules |
| PA2411 | 2.4E-09 | -2.25 | -6.7 | Adaptation, Protection; Putative enzymes |
| PA2412 | 2.6E-29 | -2.06 |  | Hypothetical, unclassified, unknown |
| PA2413_pvdH | 6.0E-25 | -1.85 |  | Adaptation, Protection |
| PA2424_pvdL | 1.6E-18 | -2.08 |  | Adaptation, Protection |
| PA2425_pvdG | 1.1E-13 | -2.27 |  | Adaptation, Protection |
| PA2426_pvdS | 9.2E-14 | -1.86 |  | Transcriptional regulators |
| PA2427 | 9.5E-22 | -2.66 |  | Hypothetical, unclassified, unknown |
| PA2433 | 2.1E-226 | -3.52 |  | Hypothetical, unclassified, unknown |
| PA2444_glyA2 | 1.3E-20 | 1.50 |  | Amino acid biosynthesis and metabolism |
| PA2446_gcvH2 | 1.3E-41 | 1.56 |  | Amino acid biosynthesis and metabolism |
| PA2462 | 1.2E-173 | 2.92 | 3.6 | Hypothetical, unclassified, unknown |
| PA2463 | 1.0E-46 | 2.59 |  | Hypothetical, unclassified, unknown |
| PA2481 | 5.5E-54 | 1.88 | 2.4 | Hypothetical, unclassified, unknown |
| PA2503 | 6.1E-29 | 1.74 |  | Hypothetical, unclassified, unknown |
| PA2504 | 5.0E-91 | -2.72 | -3.1 | Hypothetical, unclassified, unknown |
| PA2538 | 6.5E-29 | 1.99 | 1.8 | Membrane proteins |
| PA2539 | 3.6E-39 | 1.88 | 2.4 | Membrane proteins |
| PA2564 | 6.3E-49 | -1.91 | -2.7 | Hypothetical, unclassified, unknown |
| PA2565 | 4.9E-57 | -1.93 | -2.4 | Hypothetical, unclassified, unknown |
| PA2566 | 4.2E-98 | -2.10 | -17.7 | Hypothetical, unclassified, unknown |
| PA2567 | 9.4E-33 | 1.52 | 1.7 | Hypothetical, unclassified, unknown |
| PA2570_lecA | 2.8E-72 | -2.39 | -20.3 | Adaptation, Protection; Motility & Attachment; Cell wall / LPS / capsule |
| PA2571 | 3.8E-164 | -3.07 | -5.8 | Two-component regulatory systems |
| PA2572 | 1.4E-40 | -1.69 | -13.2 | Transcriptional regulators; Two-component regulatory systems |
| PA2573 | 1.4E-74 | -1.95 | -12.1 | Adaptation, Protection; Chemotaxis |
| PA2658 | 3.9E-29 | -1.57 | -1.9 | Hypothetical, unclassified, unknown |
| PA2700_opdB | 6.5E-23 | -1.86 | -3.1 | Membrane proteins; Transport of small molecules |
| PA2701 | 3.7E-33 | -1.92 | -12.3 | Membrane proteins; Transport of small molecules |
| PA2717_cpo | 2.2E-50 | -2.06 | -5.3 | Central intermediary metabolism |
| PA2721 | 9.9E-35 | -2.06 | -6.9 | Hypothetical, unclassified, unknown |
| PA2722 | 4.0E-29 | -1.94 | -3.8 | Hypothetical, unclassified, unknown |
| PA2727 | 2.0E-41 | 1.84 | 2.3 | Hypothetical, unclassified, unknown |
| PA2728 | 3.3E-32 | 1.64 | 1.8 | Hypothetical, unclassified, unknown |
| PA2729 | 1.4E-35 | 2.00 | 1.9 | Hypothetical, unclassified, unknown |
| PA2746 | 1.0E-53 | -2.23 | -7.6 | Membrane proteins |
| PA2747 | 5.7E-219 | -4.42 |  | Hypothetical, unclassified, unknown |
| PA2771 | 1.8E-36 | -1.88 | -11.6 | Hypothetical, unclassified, unknown |
| PA2787_cpg2 | 2.1E-29 | -1.62 | -2.5 | Central intermediary metabolism |
| PA2791 | 1.1E-05 | 1.86 |  | Hypothetical, unclassified, unknown |
| PA2792 | 1.6E-21 | 2.21 | 2 | Hypothetical, unclassified, unknown |
| PA2816 | 8.3E-35 | -1.99 | -2 | Hypothetical, unclassified, unknown |
| PA2817 | 2.8E-53 | -2.00 |  | Hypothetical, unclassified, unknown |
| PA2843 | 1.6E-11 | 1.55 |  | Putative enzymes |
| PA2878 | 1.2E-04 | -1.72 |  | Hypothetical, unclassified, unknown |
| PA2916 | 2.9E-17 | 1.75 | 1.8 | Membrane proteins |
| PA2927 | 9.4E-56 | -1.91 | -4.2 | Hypothetical, unclassified, unknown |
| PA2937 | 9.1E-44 | -2.18 | -8.4 | Hypothetical, unclassified, unknown |
| PA2939 | 2.3E-117 | -3.13 | -137.4 | Secreted Factors (toxins, enzymes, alginate) |
| PA3049_rmf | 2.1E-31 | -1.77 |  | Translation, post-translational modification, degradation |
| PA3055 | 4.4E-38 | -1.75 |  | Hypothetical, unclassified, unknown |
| PA3091 | 6.4E-40 | -1.62 | -2 | Hypothetical, unclassified, unknown |
| PA3182_pgl | 1.0E-22 | 1.56 |  | Central intermediary metabolism |
| PA3183_zwf | 6.5E-36 | 1.55 |  | Energy metabolism; Carbon compound catabolism |
| PA3186_oprB | 4.9E-44 | 2.19 |  | Transport of small molecules |
| PA3187 | 1.2E-62 | 2.52 |  | Transport of small molecules |
| PA3188 | 8.3E-47 | 2.64 |  | Transport of small molecules |
| PA3189 | 3.1E-37 | 2.42 |  | Transport of small molecules |
| PA3190 | 6.8E-34 | 1.95 |  | Transport of small molecules |
| PA3216 | 2.1E-27 | -1.85 | -3.3 | Membrane proteins |
| PA3250 | 3.5E-64 | -2.00 | -11.5 | Hypothetical, unclassified, unknown |
| PA3251 | 1.2E-28 | -2.04 | -7.9 | Hypothetical, unclassified, unknown |
| PA3253 | 4.8E-12 | -1.60 | -4 | Membrane proteins; Transport of small molecules |
| PA3287 | 2.0E-05 | 2.07 | -4.6 | Hypothetical, unclassified, unknown |
| PA3292 | 2.5E-49 | 3.67 | 4.7 | Hypothetical, unclassified, unknown |
| PA3293 | 6.2E-95 | 3.60 | 3.9 | Hypothetical, unclassified, unknown |
| PA3316 | 5.9E-51 | -1.67 | -2.7 | Membrane proteins; Transport of small molecules |
| PA3327 | 3.8E-47 | 2.64 | 4.3 | Adaptation, Protection |
| PA3328 | 2.2E-100 | 3.09 | 5.2 | Putative enzymes |
| PA3329 | 2.4E-117 | 3.12 | 4.8 | Hypothetical, unclassified, unknown |
| PA3330 | 8.4E-119 | 3.02 | 5.4 | Putative enzymes |
| PA3331 | 7.2E-127 | 3.09 | 3.8 | Adaptation, Protection; Carbon compound catabolism |
| PA3332 | 5.2E-130 | 3.12 | 3.9 | Hypothetical, unclassified, unknown |
| PA3333_fabH2 | 2.7E-134 | 3.03 | 3.8 | Fatty acid and phospholipid metabolism |
| PA3334 | 3.5E-101 | 3.03 | 4.9 | Fatty acid and phospholipid metabolism |
| PA3335 | 3.1E-103 | 2.90 | 4.1 | Hypothetical, unclassified, unknown |
| PA3336 | 1.5E-87 | 3.04 | 3.4 | Membrane proteins; Transport of small molecules |
| PA3340 | 2.3E-77 | -2.51 | -3.6 | Membrane proteins |
| PA3354 | 1.6E-51 | -1.68 |  | Hypothetical, unclassified, unknown |
| PA3361_lecB | 2.3E-31 | 1.77 | 1.7 | Motility & Attachment |
| PA3371 | 4.2E-39 | -1.57 |  | Hypothetical, unclassified, unknown |
| PA3391_nosR | 6.7E-16 | 1.76 |  | Membrane proteins; Energy metabolism |
| PA3392_nosZ | 5.7E-07 | 1.94 | 2.1 | Energy metabolism |
| PA3393_nosD | 2.6E-18 | 1.95 |  | Energy metabolism |
| PA3394_nosF | 2.4E-25 | 2.15 |  | Energy metabolism; Transport of small molecules |
| PA3395_nosY | 4.7E-31 | 2.59 |  | Membrane proteins; Energy metabolism |
| PA3396_nosL | 1.4E-30 | 2.43 |  | Energy metabolism |
| PA3407_hasAp | 9.1E-17 | -1.58 |  | Transport of small molecules |
| PA3415 | 2.2E-49 | -2.31 | -7.3 | Energy metabolism |
| PA3416 | 2.8E-49 | -2.16 | -16.9 | Energy metabolism |
| PA3417 | 4.8E-29 | -1.75 | -7.9 | Energy metabolism |
| PA3426 | 1.4E-38 | -1.58 | -3 | Putative enzymes |
| PA3441 | 1.5E-10 | 1.77 |  | Transport of small molecules |
| PA3451 | 8.3E-19 | -1.99 | -35.7 | Hypothetical, unclassified, unknown |
| PA3452_mqoA | 1.8E-22 | -1.68 | -3.8 | Central intermediary metabolism; Energy metabolism |
| PA3459 | 3.4E-43 | -1.92 |  | Amino acid biosynthesis and metabolism |
| PA3460 | 9.0E-32 | -1.65 |  | Putative enzymes |
| PA3461 | 1.0E-41 | -1.85 |  | Hypothetical, unclassified, unknown |
| PA3462 | 4.4E-34 | -1.50 | -3.5 | Two-component regulatory systems |
| PA3531_bfrB | 2.3E-26 | 1.51 |  | Transport of small molecules; Adaptation, Protection |
| PA3581_glpF | 2.6E-45 | 2.08 |  | Transport of small molecules |
| PA3584_glpD | 1.4E-87 | 2.66 |  | Central intermediary metabolism; Energy metabolism |
| PA3596 | 1.4E-37 | -1.97 | -2.9 | DNA replication, recombination, modification and repair |
| PA3622_rpoS | 4.5E-170 | -4.24 |  | Transcriptional regulators |
| PA3623 | 5.7E-30 | -1.81 |  | Hypothetical, unclassified, unknown |
| PA3661 | 4.4E-03 | 1.54 |  | Hypothetical, unclassified, unknown |
| PA3662 | 2.2E-62 | 2.73 | 5.7 | Hypothetical, unclassified, unknown |
| PA3688 | 7.3E-35 | -1.71 | -12.9 | Hypothetical, unclassified, unknown |
| PA3723 | 1.4E-41 | -1.57 | -9.3 | Putative enzymes |
| PA3727 | 3.4E-35 | 2.05 | 2.6 | Hypothetical, unclassified, unknown |
| PA3795 | 7.3E-89 | -2.38 |  | Putative enzymes |
| PA3858 | 7.5E-76 | -2.14 | -4 | Transport of small molecules |
| PA3871 | 1.1E-13 | 1.58 |  | Translation, post-translational modification, degradation; Chaperones & heat shock proteins |
| PA3872_narI | 4.4E-14 | 2.08 |  | Energy metabolism |
| PA3873_narJ | 5.1E-19 | 2.26 | 2 | Energy metabolism |
| PA3874_narH | 2.1E-09 | 2.32 |  | Energy metabolism |
| PA3875_narG | 7.7E-19 | 3.32 | 2.4 | Energy metabolism |
| PA3876_narK2 | 4.0E-73 | 4.28 |  | Membrane proteins; Transport of small molecules |
| PA3877_narK1 | 1.1E-89 | 4.43 |  | Membrane proteins; Transport of small molecules |
| PA3901_fecA | 3.4E-08 | 1.55 |  | Membrane proteins; Transport of small molecules |
| PA3905 | 1.6E-40 | 1.56 |  | Hypothetical, unclassified, unknown |
| PA3906 | 4.1E-64 | 2.34 |  | Hypothetical, unclassified, unknown |
| PA3907 | 6.0E-88 | 2.56 |  | Hypothetical, unclassified, unknown |
| PA3908 | 2.1E-76 | 2.77 |  | Hypothetical, unclassified, unknown |
| PA3913 | 2.8E-07 | 1.94 |  | Putative enzymes |
| PA3914_moeA1 | 5.3E-100 | 4.47 |  | Biosynthesis of cofactors, prosthetic groups and carriers |
| PA3915_moaB1 | 1.3E-64 | 3.31 | 2.3 | Biosynthesis of cofactors, prosthetic groups and carriers |
| PA3938 | 1.2E-23 | 1.56 |  | Transport of small molecules |
| PA3957 | 1.1E-30 | -1.55 | -3 | Putative enzymes |
| PA3974_ladS | 7.9E-54 | -1.60 |  | Two-component regulatory systems |
| PA3986 | 5.4E-70 | -1.97 | -5.5 | Hypothetical, unclassified, unknown |
| PA4041 | 2.7E-39 | -1.78 | -10.9 | Putative enzymes |
| PA4112 | 7.4E-42 | -1.63 | -5.8 | Two-component regulatory systems |
| PA4159_fepB | 2.3E-12 | -1.52 |  | Transport of small molecules |
| PA4166 | 1.1E-25 | 1.96 |  | Putative enzymes |
| PA4172 | 7.9E-22 | -1.65 |  | DNA replication, recombination, modification and repair |
| PA4210_phzA1 | 6.7E-12 | 1.75 |  | Secreted Factors (toxins, enzymes, alginate) |
| PA4211_phzB1 | 1.3E-36 | 1.82 | 1.5 | Secreted Factors (toxins, enzymes, alginate) |
| PA4212_phzC1 | 8.8E-20 | 1.70 |  | Secreted Factors (toxins, enzymes, alginate) |
| PA4216_phzG1 | 3.5E-27 | 2.99 |  | Secreted Factors (toxins, enzymes, alginate) |
| PA4217_phzS | 1.0E-105 | 2.98 | 2.4 | Putative enzymes |
| PA4227_pchR | 1.7E-20 | -1.79 |  | Transcriptional regulators |
| PA4228_pchD | 4.7E-43 | -2.64 |  | Secreted Factors (toxins, enzymes, alginate); Transport of small molecules |
| PA4229_pchC | 1.1E-48 | -2.65 |  | Secreted Factors (toxins, enzymes, alginate); Transport of small molecules |
| PA4230_pchB | 1.8E-41 | -2.38 |  | Secreted Factors (toxins, enzymes, alginate); Transport of small molecules |
| PA4231_pchA | 1.4E-29 | -2.40 |  | Secreted Factors (toxins, enzymes, alginate); Transport of small molecules |
| PA4290 | 9.1E-47 | -2.04 |  | Adaptation, Protection; Chemotaxis |
| PA4293_pprA | 8.1E-106 | -2.66 | -11.5 | Two-component regulatory systems |
| PA4294 | 1.8E-116 | -2.69 | -16.1 | Hypothetical, unclassified, unknown |
| PA4297_tadG | 8.3E-105 | -2.40 | -12.9 | Membrane proteins; Motility & Attachment |
| PA4298 | 3.0E-62 | -2.36 | -21.9 | Hypothetical, unclassified, unknown |
| PA4299_tadD | 6.5E-64 | -2.51 | -13.2 | Motility & Attachment |
| PA4300_tadC | 4.0E-93 | -2.50 | -16.7 | Membrane proteins; Motility & Attachment |
| PA4301_tadB | 1.7E-101 | -2.72 | -7.3 | Membrane proteins; Motility & Attachment |
| PA4302_tadA | 4.9E-154 | -3.21 | -26.2 | Protein secretion/export apparatus; Motility & Attachment |
| PA4303_tadZ | 2.8E-115 | -3.28 | -12 | Motility & Attachment |
| PA4304_rcpA | 7.4E-174 | -3.54 | -21.7 | Protein secretion/export apparatus; Motility & Attachment |
| PA4305_rcpC | 2.3E-93 | -3.35 | -11.2 | Motility & Attachment |
| PA4306_flp | 2.9E-24 | -1.99 | -76 | Motility & Attachment |
| PA4317 | 2.0E-14 | 1.74 | 1.8 | Membrane proteins |
| PA4319 | 6.4E-36 | 1.69 |  | Membrane proteins |
| PA4321 | 2.6E-36 | 2.00 |  | Hypothetical, unclassified, unknown |
| PA4322 | 1.4E-42 | 1.92 |  | Hypothetical, unclassified, unknown |
| PA4323 | 8.5E-53 | 1.92 |  | Hypothetical, unclassified, unknown |
| PA4362 | 1.0E-39 | -1.77 | -9.2 | Hypothetical, unclassified, unknown |
| PA4377 | 2.6E-58 | -3.52 |  | Hypothetical, unclassified, unknown |
| PA4467 | 6.4E-09 | -2.52 |  | Membrane proteins |
| PA4468_sodM | 3.1E-10 | -2.72 |  | Adaptation, Protection |
| PA4469 | 3.2E-08 | -2.69 |  | Hypothetical, unclassified, unknown |
| PA4470_fumC1 | 1.8E-06 | -2.33 |  | Energy metabolism |
| PA4471 | 4.8E-04 | -1.94 |  | Hypothetical, unclassified, unknown |
| PA4498 | 1.2E-47 | -1.59 | 1.7 | Translation, post-translational modification, degradation |
| PA4570 | 1.6E-86 | -3.34 |  | Hypothetical, unclassified, unknown |
| PA4572_fklB | 1.1E-49 | -1.92 | -1.7 | Translation, post-translational modification, degradation; Chaperones & heat shock proteins |
| PA4573 | 2.9E-91 | -2.55 | -10.7 | Hypothetical, unclassified, unknown |
| PA4578 | 1.3E-31 | 1.81 | 1.7 | Hypothetical, unclassified, unknown |
| PA4587_ccpR | 2.1E-28 | 1.63 | 2.9 | Energy metabolism |
| PA4607 | 2.5E-171 | -3.52 | -5.1 | Hypothetical, unclassified, unknown |
| PA4610 | 3.7E-79 | 2.12 |  | Hypothetical, unclassified, unknown |
| PA4785 | 7.3E-30 | -1.57 | -2.4 | Putative enzymes |
| PA4822 | 1.4E-28 | -4.30 |  | Membrane proteins |
| PA4823 | 3.8E-92 | -4.56 |  | Hypothetical, unclassified, unknown |
| PA4824 | 7.0E-128 | -5.00 |  | Hypothetical, unclassified, unknown |
| PA4825_mgtA | 2.1E-242 | -5.45 |  | Transport of small molecules |
| PA4829_lpd3 | 1.3E-38 | -1.70 | -3.7 | Energy metabolism |
| PA4874 | 6.1E-43 | -1.73 | -7.5 | Hypothetical, unclassified, unknown |
| PA4905_vanB | 2.0E-11 | -1.67 |  | Carbon compound catabolism |
| PA4925 | 4.2E-41 | -1.82 | -18.8 | Hypothetical, unclassified, unknown |
| PA4929 | 6.9E-39 | -1.60 | -4.7 | Membrane proteins |
| PA5024 | 7.5E-10 | 1.52 |  | Hypothetical, unclassified, unknown |
| PA5026 | 3.4E-29 | 1.69 |  | Hypothetical, unclassified, unknown |
| PA5058_phaC2 | 2.8E-111 | -2.65 | -13.5 | Central intermediary metabolism |
| PA5059 | 2.0E-125 | -2.91 | -13.6 | Transcriptional regulators |
| PA5087 | 1.3E-10 | 1.52 |  | Hypothetical, unclassified, unknown |
| PA5088 | 1.2E-29 | 2.13 |  | Hypothetical, unclassified, unknown |
| PA5101 | 9.4E-66 | -1.92 | -9.3 | Hypothetical, unclassified, unknown |
| PA5113 | 5.5E-84 | 2.34 | 2.4 | Membrane proteins |
| PA5136 | 8.9E-57 | 1.98 | 1.7 | Hypothetical, unclassified, unknown |
| PA5213_gcvP1 | 2.8E-52 | -2.11 | -7.3 | Central intermediary metabolism; Amino acid biosynthesis and metabolism |
| PA5265 | 9.1E-42 | 2.00 | 3.8 | Membrane proteins |
| PA5267_hcpB | 1.0E-77 | 4.27 |  | Secreted Factors (toxins, enzymes, alginate) |
| PA5314 | 3.5E-36 | -2.01 |  | Hypothetical, unclassified, unknown |
| PA5352 | 1.1E-115 | 3.24 | 4.3 | Hypothetical, unclassified, unknown |
| PA5353_glcF | 1.1E-159 | 3.28 |  | Central intermediary metabolism; Carbon compound catabolism |
| PA5354_glcE | 1.3E-119 | 3.05 | 3.5 | Central intermediary metabolism; Carbon compound catabolism |
| PA5355_glcD | 4.0E-75 | 2.71 |  | Central intermediary metabolism; Carbon compound catabolism |
| PA5359 | 5.8E-57 | -2.17 | -6.5 | Hypothetical, unclassified, unknown |
| PA5372_betA | 1.1E-34 | 1.63 |  | Amino acid biosynthesis and metabolism; Adaptation, Protection |
| PA5373_betB | 1.1E-51 | 2.19 |  | Amino acid biosynthesis and metabolism; Adaptation, Protection |
| PA5374_betI | 2.8E-19 | 1.94 |  | Transcriptional regulators |
| PA5420_purU2 | 8.3E-22 | 1.53 |  | Nucleotide biosynthesis and metabolism |
| PA5436 | 9.3E-34 | 1.54 |  | Central intermediary metabolism |
| PA5460 | 3.8E-10 | 1.57 |  | Hypothetical, unclassified, unknown |
| PA5530 | 2.7E-08 | 2.00 |  | Membrane proteins; Transport of small molecules |

**References**

Han, K., Tjaden, B., and Lory, S. (2016). GRIL-seq provides a method for identifying direct targets of bacterial small regulatory RNA by in vivo proximity ligation. *Nat Microbiol* 2**,** 16239.

Heeb, S., Itoh, Y., Nishijyo, T., Schnider, U., Keel, C., Wade, J., Walsh, U., O'gara, F., and Haas, D. (2000). Small, stable shuttle vectors based on the minimal pVS1 replicon for use in gram-negative, plant-associated bacteria. *Mol Plant Microbe Interact* 13**,** 232-237.

Holloway, B.W., Krishnapillai, V., and Morgan, A.F. (1979). Chromosomal genetics of *Pseudomonas*. *Microbiol Rev* 43**,** 73-102.

Norrander, J., Kempe, T., and Messing, J. (1983). Construction of improved M13 vectors using oligodeoxynucleotide-directed mutagenesis. *Gene* 26**,** 101-106.

Schuster, M., Hawkins, A.C., Harwood, C.S., and Greenberg, E.P. (2004). The *Pseudomonas aeruginosa* RpoS regulon and its relationship to quorum sensing. *Mol Microbiol* 51**,** 973-985.

Simon, R., O'connell, M., Labes, M., and Puhler, A. (1986). Plasmid vectors for the genetic analysis and manipulation of rhizobia and other gram-negative bacteria. *Methods Enzymol* 118**,** 640-659.

Simon, R., Priefer, U., and Pühler, A. (1983). A Broad Host Range Mobilization System for *In Vivo* Genetic Engineering: Transposon Mutagenesis in Gram Negative Bacteria. *Bio/Technology* 1**,** 784-791.

Schneider-Keel, U., Seematter, A., Maurhofer, M., Blumer, C., Duffy, B., Gigot-Bonnefoy,

C., Reimmann, C., Notz, R., Défago, G., Haas, D., and Keel, C. (2000). Autoinduction of2,4-Diacetylphloroglucinol Biosynthesis in the Biocontrol Agent *Pseudomonas fluorescens* CHA0 and Repression by the Bacterial Metabolites Salicylate and Pyoluteorin. *J Bacteriol* 182**,** 1215-1225.

Sonnleitner, E., Prindl, K., and Bläsi, U. (2017). The *Pseudomonas aeruginosa* CrcZ RNA interferes with Hfq-mediated riboregulation. *PLoS One* 12(7)**,** e0180887. doi: 10.1371/journal.pone.0180887.

Suh, S.J., Silo-Suh, L., Woods, D.E., Hassett, D.J., West, S.E., and Ohman, D.E. (1999). Effect of *rpoS* mutation on the stress response and expression of virulence factors in *Pseudomonas aeruginosa*. *J Bacteriol* 181**,** 3890-3897.
